# A Trehalose-Based Phenotypic Screen Identifies Candidate Inhibitors of *Mycobacterium tuberculosis* Recycling Pathway

**DOI:** 10.3390/antibiotics15080743

**Published:** 2026-07-31

**Authors:** Rebecca Vande Voorde, Aaron M. Maves, Dylan Nelson, Lia Danelishvili

**Affiliations:** 1 Department of Biomedical Sciences, Carlson College of Veterinary Medicine, Oregon State University, Corvallis, OR 97331, USA; 2 Department of Microbiology, College of Science, Oregon State University, Corvallis, OR 97331, USA; 3 Department of Pharmaceutical Sciences, College of Pharmacy, Oregon State University, Corvallis, OR 97331, USA

**Keywords:** *Mycobacterium tuberculosis*, the high-throughput screen, small molecules, non-replicating, persistent, drug resistance, Trehalose recycling pathway, LpqY-SugABC transporter

## Abstract

**Background/Objectives**: Phenotypic drug tolerance, distinct from genetic resistance, allows *Mycobacterium tuberculosis* (Mtb) to survive prolonged antibiotic exposure and contributes to treatment failure and relapse. The trehalose recycling pathway, mediated by the LpqY-SugABC transporter, has been implicated as a metabolic “escape” mechanism that sustains Mtb viability under antibiotic and nutrient-limiting stress, making it an attractive target for adjunctive, tolerance-breaking therapeutics. **Methods and Results**: Here, we conducted a high-throughput phenotypic screen of 50,000 compounds from chemically diverse libraries, using a carbon source-restricted assay that forces Mtb to rely on trehalose uptake for growth, to identify small-molecule inhibitors of this pathway. This approach yielded 23 confirmed hits in Mtb, spanning several chemical scaffolds, including thioureas, propanamides, benzamides, and carboxamides. Using an isogenic set of Mtb wild-type, LpqY-SugABC transposon knockout, and complemented strains, we confirmed that the genetic loss of transporter loss reproduces accelerated killing by isoniazid, rifampicin, and bedaquiline, but not moxifloxacin, and that loss of trehalose recycling sensitizes mycobacteria to oxidative stress. Using orthogonal functional assays, fluorescent trehalose probe (FITC-tre) uptake inhibition and H_2_O_2_ hypersensitization, thiourea-containing compounds emerged as the candidates most consistent with transporter-specific activity, phenocopying the effects of genetic LpqY-SugABC loss, while biochemical assays against recombinant trehalase (Rv2402) excluded downstream enzymatic inhibition as their mechanism of action. In addition, several hits potentiated rifampicin-mediated killing of intracellular Mtb in THP-1 macrophages, in some cases reducing bacterial burden below levels achieved by monotherapy. **Conclusions**: These findings indicate that the trehalose recycling pathway is functionally druggable by small molecules identified through unbiased phenotypic screening and nominate thiourea- and propanamide-based scaffolds as priority candidates for further mechanistic characterization, including direct target-engagement studies, and optimization as adjunctive anti-tuberculosis agents targeting drug-tolerant Mtb populations.

## 1. Introduction

*Mycobacterium tuberculosis* (Mtb) is a major global pathogen, driving widespread illness and death each year while disproportionately burdening communities with limited resources [1]. Despite effective drugs, tuberculosis treatment is still prolonged and is further complicated by multidrug-resistant strains, thereby severely limiting therapeutic options [2,3]. The extended duration of therapy creates substantial barriers to treatment success, as patient adherence is often difficult to maintain [4]. In addition to these challenges, sustained exposure to sublethal drug concentrations generates selective pressure that promotes the emergence of antimicrobial resistance while also enriching persistent, drug-tolerant subpopulations of otherwise drug-susceptible bacteria [5,6,7]. This phenomenon, wherein susceptible bacteria survive lethal antibiotic concentrations through phenotypic tolerance rather than genetic mutation, represents a critical yet underappreciated mechanism of treatment failure distinct from classical antibiotic resistance [5,6,8,9].

Mtb exhibits remarkable metabolic flexibility that allows the organism to adopt alternative phenotypic states in response to hostile environmental pressures, including antibiotic exposure and the nutrient-deprived conditions of the host macrophage [10,11,12]. Upon exposure to bactericidal antibiotics, Mtb exhibits delayed killing dynamics, maintaining viability despite sustained drug pressure at concentrations exceeding inhibitory levels [11,13]. This attenuated bactericidal response points to the activation of intrinsic mechanisms that enable the bacterium to withstand antimicrobial stress. Supporting this view, proteomic analyses following treatment with diverse anti-tuberculosis agents, including isoniazid, rifampicin, moxifloxacin, and bedaquiline, reveal early induction of metabolic enzymes and transport systems associated with adaptive stress responses [12,14]. These adaptive responses are consistent with metabolic remodeling and a shift toward alternative carbon and energy utilization pathways, thereby promoting phenotypic drug tolerance [14,15]. Recent work further supports central carbon metabolic rewiring as a key contributor to Mtb drug tolerance [16,17]. Elucidation of these pathways may inform the development of combination regimens designed to concurrently inhibit primary drug targets and the compensatory mechanisms that support bacterial tolerance to antibiotics, with the potential to enhance bactericidal activity, shorten treatment duration, and limit the emergence of resistance [18,19].

The trehalose recycling pathway is a key adaptive mechanism that enables Mtb to preserve cell wall integrity and sustain energy metabolism under the nutrient-limited conditions of host macrophages [20,21]. While Mtb can utilize glucose and other abundant carbon sources in nutrient-rich culture [22], it must rely on alternative metabolic strategies in the intracellular environment, where nutrient availability is severely constrained [15]. Mtb can also use trehalose as a sole carbon and energy source, converting it to glucose through the action of trehalase [17]. This flexibility becomes especially important during stationary phase or nutrient starvation, when trehalose salvage from cell-wall turnover supports bacterial survival and persistence [17,22]. In addition, trehalose-containing glycolipids such as trehalose dimycolate (TDM) and trehalose monomycolate (TMM) are not merely structural constituents of the mycobacterial envelope but also important virulence factors that modulate host immune responses and promote intracellular survival [20]. TDM, also known as cord factor, is particularly notable for its potent immunostimulatory activity and its ability to induce granuloma formation even in the absence of viable bacteria, underscoring the central role of trehalose metabolism in Mtb pathogenesis [17,20]. Recent structural and functional studies have also shown that trehalose recycling promotes energy-efficient biosynthesis of the mycobacterial cell envelope [7,8,10,12,23].

Recent proteomic studies by our group and others indicate that the LpqY-SugABC transporter is upregulated following antibiotic exposure, suggesting that Mtb responds to drug-induced metabolic stress by enhancing trehalose scavenging and recycling during cell wall remodeling [14,17,24,25]. Functional disruption of this system has been shown to accelerate killing by isoniazid, rifampicin, and bedaquiline in vitro and in infected human macrophages, supporting a role for trehalose recycling in antibiotic tolerance and survival [17,26]. Accordingly, the LpqY-SugABC system represents an attractive therapeutic target because it contributes to in vivo virulence and persistence, is induced under antibiotic pressure, may be accessible to cell surface-directed inhibitors, and is absent from mammalian cells, reducing the likelihood of host toxicity [17]. Structural studies have further clarified trehalose recognition, capture, and translocation by LpqY-SugABC, strengthening its appeal as a drug target [8,9,10,12,24,25]. Prior chemical approaches to exploiting this transporter have proceeded along two distinct strategies. First, synthetic trehalose analogues have been used as carbohydrate-based antimicrobial and anti-biofilm agents whose activity in *M. smegmatis* was shown to depend on LpqY-SugABC-mediated cellular uptake, demonstrating that this transporter can serve as a delivery route for chemical cargo into mycobacteria [27]; however, this approach reported only moderate potency, was restricted to a non-pathogenic surrogate species, and was not evaluated against Mtb or within an intracellular host-cell environment. A separate effort to identify small-molecule ligands of the transporter screened the curated, ~400-compound Medicines for Malaria Venture Pathogen Box using a biophysical, binding-based approach, and identified seven structurally diverse compounds that recognize LpqY [26]. However, this screen was restricted to a small, pre-selected chemical library, and was designed to detect direct binding to LpqY rather than functional inhibition of transporter-mediated trehalose uptake or bacterial growth; and did not assess whole-cell antibacterial activity, carbon source-dependent phenotypic effects, or efficacy against intracellular Mtb within host macrophages. Collectively, these prior studies establish that LpqY-SugABC can be chemically engaged, either as a route for carbohydrate-conjugate delivery [27] or as a target for direct ligand binding [26], but neither approach has identified compounds that functionally inhibit transporter-mediated trehalose recycling in intact, physiologically relevant Mtb, nor translated such inhibition into potentiation of existing anti-TB drugs within the host-cell environment. A large-scale, unbiased phenotypic screen capable of addressing this gap has not previously been reported.

To identify small-molecule inhibitors of the LpqY-SugABC transporter that could potentiate the activity of existing anti-tuberculosis drugs, we employed a phenotypic screening approach centered on trehalose metabolism under nutrient-deprived conditions, using a bioavailable trehalose probe, selectively recognized and transported by the LpqY-SugABC system [28]. By allowing real-time monitoring of trehalose recycling in living cells, this strategy facilitates the identification of compounds that disrupt transporter function [28,29,30]. More importantly, this approach reflects the metabolic state encountered by intracellular bacteria and enables the identification of compounds that impair trehalose recycling in a physiologically relevant stress environment [28]. Because trehalose recycling supports survival during carbon limitation and is linked to envelope maintenance and stress adaptation, inhibitors of this pathway may weaken intracellular bacterial fitness, attenuate persistent phenotypes, and enhance the activity of standard anti-TB drugs [17,22].

In the present study, we conducted a high-throughput screen of a chemically diverse compound library to identify inhibitors of the LpqY-SugABC trehalose recycling pathway. Selected hits were then structurally characterized, evaluated for potency and selectivity, assessed for synergistic bactericidal activity in combination with standard anti-TB drugs in macrophage infection models, and examined for their effects on trehalose metabolism. Identification of compounds that block trehalose recycling may provide a basis for rational combination therapies designed to accelerate Mtb killing, shorten treatment duration, and reduce the emergence of drug resistance.

## 2. Results

### 2.1. Identification of Compounds That Selectively Inhibit Mycobacterial Growth on Trehalose

**Figure 1:**
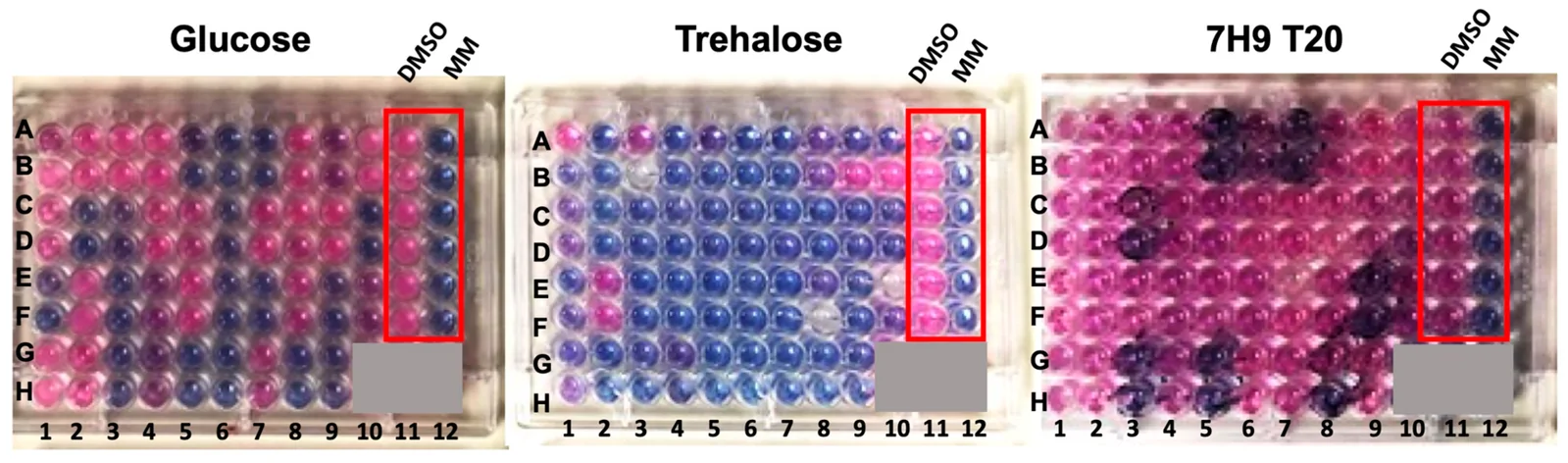
Resazurin-based metabolic assay identifying trehalose-selective growth inhibitors in Mtb. Three 96-well plates showing the resazurin-based metabolic assay performed as described in the Section 4. The plates display results for the *Mtb* wild-type strain tested against 39 candidate compounds, arranged in columns 1–9 of rows A–H and in column 10 of rows A–F. Each compound was tested in duplicate wells within the same column. Each plate represents a different carbon source condition: glucose (MM + 20 mM glucose), trehalose (MM + 10 mM trehalose), and 7H9 T20 (7H9 broth + Tween 20). Pink/magenta wells indicate metabolic activity and bacterial growth (resazurin reduced to the fluorescent product resorufin); blue/dark purple wells indicate growth inhibition or metabolic inactivity (unreduced resazurin). The right columns (red-boxed areas) show control wells: DMSO (vehicle control wells in column 11 of rows A-F containing DMSO alone in each medium, indicating maximum growth for that condition) and MM (minimal medium in column 12 of rows A–F without carbon source, showing no growth or color change, as expected for the negative control).

We designed an HTS assay in 384-well plate format to identify compounds that inhibited the ability of *M. smegmatis* mc^2^155 to utilize trehalose as a carbon source during growth in minimal medium. Potential hits were identified as compounds that prevented or slowed bacterial growth, as indicated by inhibition of the resazurin color change (blue to pink, reflecting reduction to the fluorescent product resorufin by metabolically active cells) relative to DMSO-alone control wells. Plate fluorescence was measured on a Tecan plate reader, and plates were also visually inspected to identify compounds that inhibited growth in trehalose-containing medium by more than 50%. A total of 50,000 compounds were tested in the primary HTS assay, yielding 168 potential hits that prevented resazurin reduction. These hits were evaluated in a secondary counter-screen comparing *M. smegmatis* mc^2^155 growth in MM supplemented with 10 mM trehalose to growth in MM supplemented with 20 mM glucose and 7H9 broth supplemented with Tween 20. Compounds that inhibited growth in all tested media were classified as general growth inhibitors rather than trehalose pathway-specific inhibitors and were excluded from further analysis. Of the 168 compounds tested, 57 selectively inhibited the growth of *M. smegmatis* mc^2^155 in trehalose-supplemented MM while exhibiting little or no effect on growth in glucose-supplemented MM or 7H9 broth. These 57 compounds were reordered from the vendor and initially retested in *M. smegmatis*. Of these, 39 compounds with confirmed activity were subsequently evaluated in Mtb using a similar experimental approach (Figure 1). Growth inhibition scoring was corroborated by concordant fluorescence and colorimetric readouts of resazurin reduction across all screening plates, and the clear, non-overlapping separation between maximal-growth (DMSO) and maximal-inhibition (no-carbon MM) control wells (Figure 1) supported a robust assay window. Hits were defined as compounds producing greater than 50% inhibition of resazurin reduction relative to DMSO controls, as confirmed by both fluorescence and colorimetric assessment. The primary screen achieved a Z′-factor and signal-to-background ratio consistent with a reliable HTS assay and yielded an overall hit rate of 168/50,000 compounds (0.34%), consistent with hit rates typically reported for phenotypic anti-mycobacterial screening campaigns [29,31].

Twenty-three of 39 compounds reproducibly inhibited Mtb growth in trehalose-containing medium but not in glucose supplemented or 7H9 media and are listed in the Table 1. These confirmed hits represented several structurally distinct chemical classes, including propanamides, thioureas, benzamides, and carboxamides, among others.

### 2.2. Cytotoxicity of LpqY-SugABC Inhibitor Candidates

The cytotoxicity of 23 compounds was assessed in differentiated THP-1 macrophages and A549 alveolar epithelial cells, as well as in minimal medium conditions supplemented with trehalose as the sole carbon source using the resazurin cell viability assay (Table 2). Compounds were evaluated in the concentration range of 0.1–100 μM using a resazurin colorimetric assay coupled with visual microscopic examination of macrophage monolayers. Of the 23 compounds tested, 13 compounds (57%) demonstrated no detectable toxicity to mammalian cells, while 10 compounds (43%) exhibited cytotoxicity at 100 μM. Among the non-toxic compounds, significant variation in the ability to inhibit trehalose-dependent bacterial growth was observed. Six compounds (**59**, **60**, **66**, **72**, **82**, and **95**) with IC_50_ values of 3–10 μM demonstrated potent inhibition of Mtb growth and complete absence of mammalian cell toxicity. Four additional compounds (**67**, **76**, **86**, and **88**) showed moderate inhibitory potency (IC_50_ of 32 μM) while remaining non-toxic to macrophages. Three compounds (**75**, **80**, and **83**) exhibited weaker inhibition (IC_50_ of 100 μM) but maintained excellent selectivity. In contrast, four compounds (**61**, **68**, **73**, and **74**) that demonstrated potent growth inhibition (IC_50_ of 3 μM in trehalose medium) exhibited intolerable cytotoxicity to both THP-1 and A549 cells at 100 μM.

### 2.3. Intracellular Activity of Compounds

**Figure 2:**
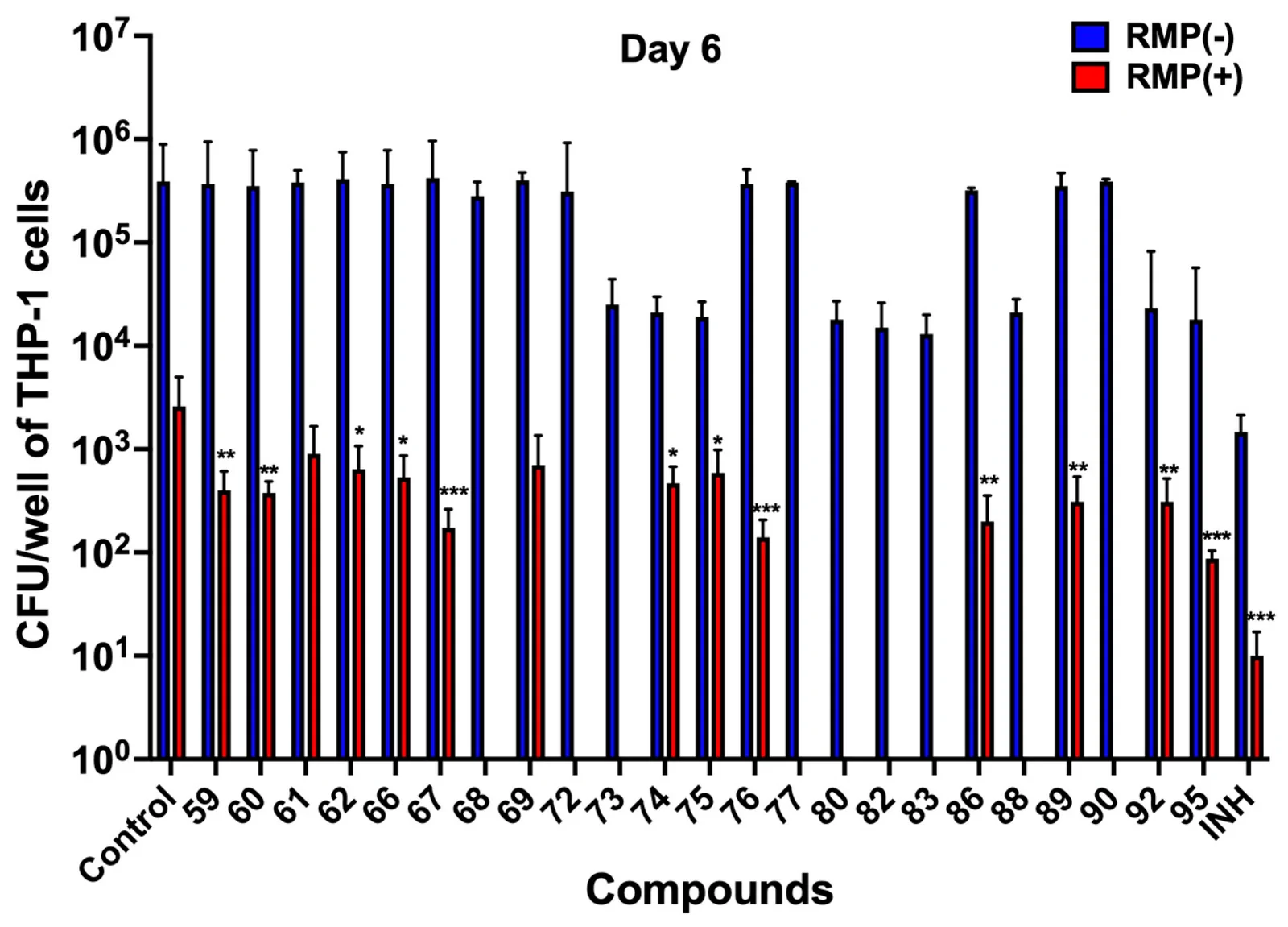
Intracellular bacterial survival and compound–antibiotic combination effect in infected macrophages. Intracellular bacterial survival assays were performed in THP-1 macrophages infected with Mtb wild type at multiplicity of infection (MOI) of 10 bacteria to 1 cell. Following 2 h infection, extracellular bacteria were removed, and infected monolayers were treated with each test compound at the highest non-toxic concentration (Table 2) without (blue bars) and/or with rifampicin (RMP, 2 μg/mL) (red bars). Infected macrophages were cultured for 6 days post-infection and then lysed with 0.1% Triton X-100, and intracellular bacteria were enumerated by serial dilution and plating on Middlebrook 7H10 agar. The CFU counts per well lysate are expressed on a log scale. DMSO-treated wells served as untreated control, while RMP and INH (2 μg/mL)-treated wells served as controls for growth inhibition. Data represent the mean ± SD of three independent biological experiments (n = 3), each performed with four technical replicates. Statistical comparisons of each compound–RMP combination-treated group relative to the RMP(+)-alone control group were performed using one-way ANOVA with Dunnett’s post hoc multiple-comparison test; * *p* < 0.05, ** *p* < 0.01, and *** *p* < 0.001.

Intracellular Mtb growth in THP-1 macrophages was evaluated following treatment with 23 selected compounds alone at their highest non-cytotoxic concentrations or in combination with RMP at subinhibitory concentration (Figure 2). In the absence of RMP treatment, DMSO-treated control wells accumulated approximately 5 × 10^5^ colony-forming units (CFU) per well by day 6 post-infection. Among the 23 test compounds, most demonstrated activity with bacterial burdens remaining comparable to the vehicle control (>1 × 10^5^ CFU/well), and nine compounds (**73**, **74**, **75**, **80**, **82**, **83**, **88**, **92**, and **95**) showed more than 1-log reductions in intracellular bacterial burden relative to untreated controls when used alone (Figure 2). In addition, when combined with RMP, most compounds produced significant reduction in intracellular Mtb burden to levels below those achieved with RMP or INH monotherapy. Notably, nine compounds (**68**, **72**, **73**, **77**, **80**, **82**, **83**, **88**, and **90**) exhibited strong activity in combination with RMP, resulting in complete bacterial clearance. The pronounced synergistic effects observed with these compounds warrant further investigation into their mechanisms of action and their potential for use in combination chemotherapy.

### 2.4. Genetic Validation of the LpqY-SugABC Transporter System

**Figure 3:**
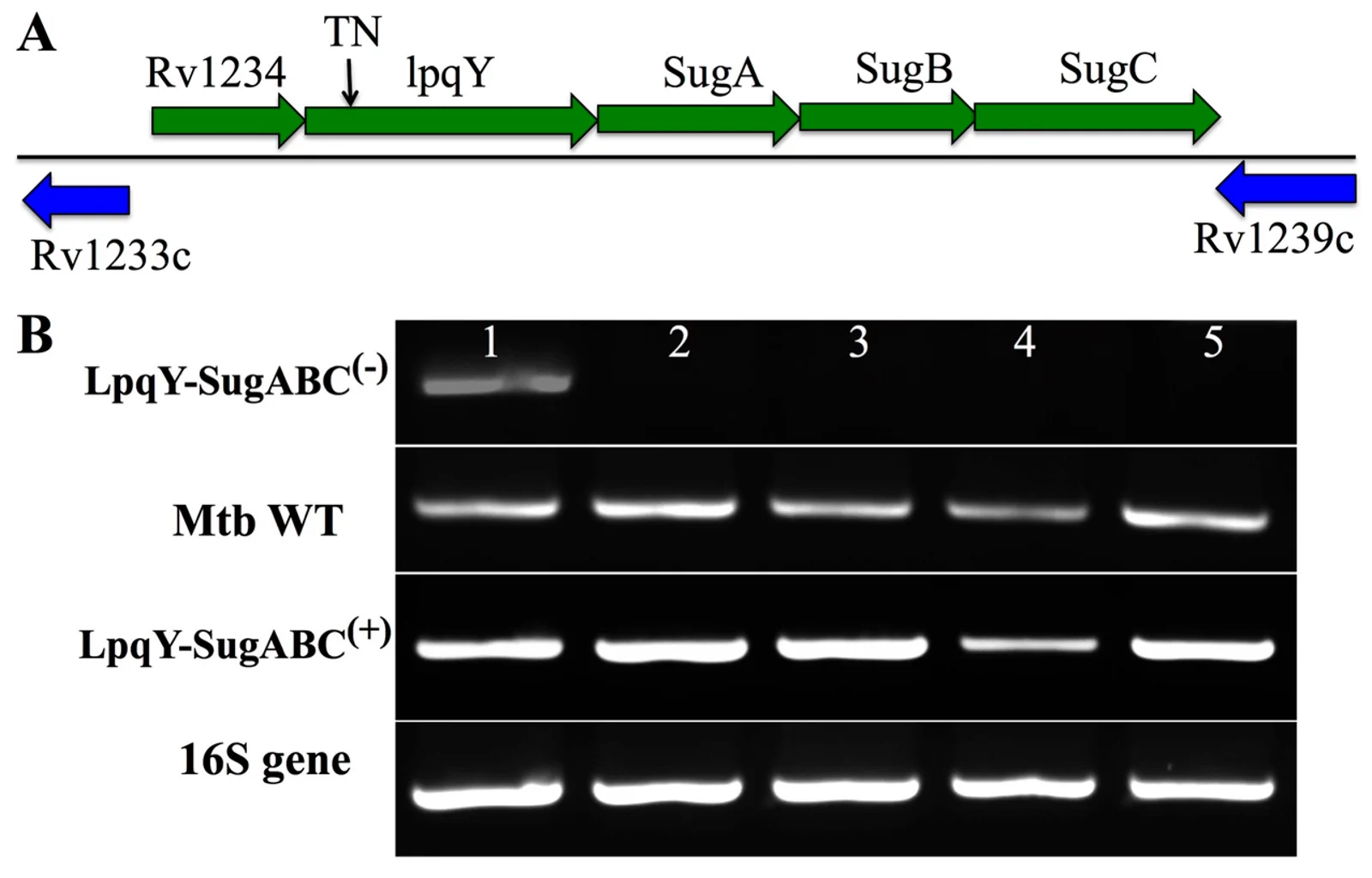
Analysis of LpqY-SugABC operon. (**A**) LpqY acts as solute binding protein, SugA and SugB are transmembrane proteins, and SugC is an ATP-hydrolyzing protein. The *Rv1234* gene encodes a probable transmembrane protein. TN—transposon insertion site. (**B**) The qualitative assessment of LpqY-SugABC transcription by RT-PCR. The total RNA samples were obtained from the LpqY-SugABC knockout, complemented, and Mtb wild type that were grown in bacterial culture medium at the mid-log phase. The RT-PCR products of ~150 bp gene products were amplified using Reverse Transcription PCR (RT-PCR). Lane 1—*Rv1234* gene; lane 2—LpqY; lane 3—SugA; lane 4—SugB; and lane 5—SugC. The housekeeping 16S was used as a reference gene.

To establish a genetic system for validating trehalose-specific transport by the LpqY-SugABC transporter, we obtained an Mtb LpqY transposon knockout mutant from BEI Resources and generated the complemented Mtb LpqY-SugABC^(+)^ strain. The LpqY-SugABC operon comprises five genes encoding the functional components of an ABC transporter: *Rv1234* (probable transmembrane protein), *lpqY* (lipoprotein solute-binding protein), *sugA* (transmembrane permease), *sugB* (transmembrane permease), and *sugC* (ATP-hydrolyzing protein) (Figure 3A). The transposon insertion site is positioned within *lpqY*, immediately downstream of *Rv1234*. To characterize the genetic organization and assess potential polar effects of the transposon insertion, we performed qualitative RT-PCR analysis on total RNA extracted from Mtb wild type, the LpqY-SugABC^(−)^ knockout strain, and the complemented LpqY-SugABC^(+)^ strain grown to mid-log phase. As illustrated in Figure 3B, transposon insertion in *lpqY* resulted in loss of detectable transcription of downstream operon genes (*lpqY*, *sugA*, *sugB*, and *sugC*), confirming strong polar effects on the operon. Notably, transcription of the upstream *Rv1234* gene remained unaffected in the knockout strain, indicating that the transposon insertion did not disrupt promoter activity. In contrast, the complemented LpqY-SugABC^(+)^ strain restored detectable transcription of the disrupted locus, demonstrating successful chromosomal integration and functional restoration of transcriptional activity across the operon. The housekeeping *16S* gene served as a loading control and confirmed equal RNA quality across all samples. These RT-PCR results validate the integrity of the knockout and complemented strains, establishing a genetic system suitable for functional investigation of LpqY-SugABC.

### 2.5. The LpqY-SugABC Transporter Mediates Drug Tolerance in Mtb-Infected Macrophages

**Figure 4:**
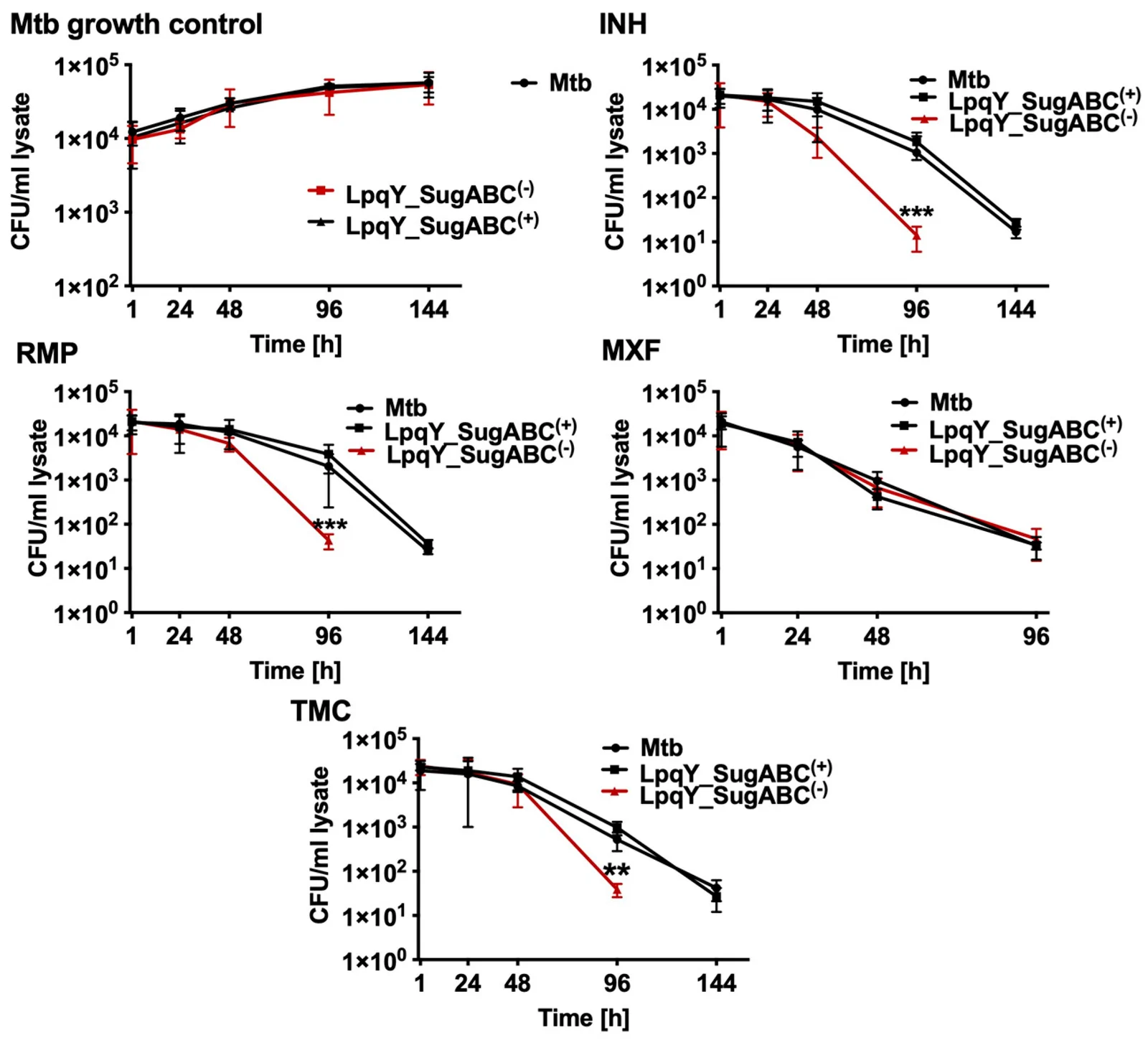
Antimicrobial susceptibility of Mtb LpqY-SugABC clones in macrophage infection assays. Mtb wild-type, LpqY-SugABC^(+)^-complemented, and LpqY-SugABC^(−)^ knockout clones were grown to mid-log phase (OD_600_ 0.4–0.6) in Middlebrook 7H9 T20 at 37 °C. Bacterial inocula were prepared at MOI 10 and used to infect THP-1 macrophage monolayers in 96-well plates for 2 h at 37 °C in 5% CO_2_. Following infection, extracellular bacteria were removed by washing with pre-warmed HBSS, and infected macrophages were treated with INH (2 μg/mL), RMP (2 μg/mL), MXF (1 μg/mL), TMC (0.12 μg/mL), or left untreated (control). Intracellular bacterial survival was assessed at multiple post-infection time points over 144 h by lysing macrophages with 0.1% Triton X-100, serially diluting lysates, and plating on Middlebrook 7H10 agar for CFU enumeration. Data represent ± SD CFU counts from three independent biological replicates (n = 3), each with three technical replicates. Statistical significance was determined using two-way ANOVA with Tukey’s post hoc multiple-comparison test at each time point; ** *p* < 0.01, and *** *p* < 0.001 for LpqY-SugABC^(−)^ vs. Mtb wild type at the corresponding time point.

To investigate the role of the LpqY-SugABC transporter in Mtb drug tolerance, we evaluated intracellular bacterial survival kinetics of Mtb wild type, the LpqY-SugABC^(−)^ knockout strain, and the complemented LpqY-SugABC^(+)^ strain in THP-1 macrophages under control and antimicrobial agent-treated conditions (Figure 4). In the absence of antimicrobial agents, all three strains exhibited indistinguishable growth kinetics, reaching approximately 10^4^–10^5^ CFU/mL lysate by 96–144 h post-infection (Figure 4, growth control). These results demonstrate that loss of the LpqY-SugABC transporter does not compromise Mtb viability under nutrient-replete conditions, thereby enabling fair assessment of drug-specific phenotypes. Notably, the LpqY-SugABC transporter played a critical role in mediating tolerance to select antimicrobial agents. Under INH, RMP, and TMC treatment, the LpqY-SugABC^(−)^ knockout strain exhibited markedly enhanced and accelerated bacterial killing by 96 h post-treatment compared with the wild type and complemented clone (Figure 4, INH, RMP, and TMC panels). This accelerated killing of the knockout mutant (red line) demonstrates loss of the drug-tolerant phenotype. In contrast, the complemented LpqY-SugABC^(+)^ strain and the wild-type control demonstrated gradual bacterial decline over time, with significant killing effects observed by 144 h post-treatment. Interestingly, susceptibility to MXF remained largely unaffected by LpqY-SugABC status, with all three strains displaying comparable killing kinetics regardless of transporter presence (Figure 4, MXF panel). Critically, complementation with LpqY-SugABC^(+)^ fully restored the drug-tolerant phenotype to near the Mtb wild-type levels across INH, RMP, and TMC, as evidenced by the similar temporal killing profiles and bacterial survival patterns (Figure 4, INH, RMP, and TMC panels). Collectively, these data establish that the LpqY-SugABC transporter is a critical determinant of Mtb tolerance to select antibiotics.

### 2.6. Loss of Trehalose Recycling Sensitizes Mycobacteria to Hydrogen Peroxide

Compounds that specifically inhibit the LpqY-SugABC trehalose transporter are predicted to reduce intracellular trehalose availability, thereby compromising antioxidant defenses and increasing bacterial susceptibility to H_2_O_2_-induced oxidative damage [23]. To evaluate the effects of trehalose recycling on ROS sensitivity, we examined H_2_O_2_ susceptibility in *M. smegmatis* and *M. tuberculosis* strains lacking functional trehalose transporter components under carbon-limiting conditions. *M. smegmatis* wild-type mc^2^155 strain, ΔsugC, and ΔlpqY mutants, along with Mtb wild type and LpqY-SugABC^(–)^ mutants, were cultured in 0.02% glucose for 24 h prior to H_2_O_2_ exposure. CFU quantification from serial dilution spot assays demonstrated that loss of trehalose recycling significantly increased susceptibility to hydrogen peroxide in both species. *M. smegmatis* ΔsugC and ΔlpqY exhibited approximately 10- and 15-fold greater sensitivity to 0.3% H_2_O_2_ than wild-type controls (Table 3). Similar results were observed in Mtb, where the LpqY-SugABC^(–)^ mutant exhibited markedly increased susceptibility with survival reduced approximately 20-fold relative to the wild type after 1 h of exposure to 0.4% H_2_O_2_. These findings indicate that the LpqY-SugABC transporter is required for resistance to oxidative stress during carbon limitation. Complementation of the mutant largely restored H_2_O_2_ resistance, confirming that disruption of trehalose recycling underlies the ROS-sensitive phenotype.

**Figure 5:**
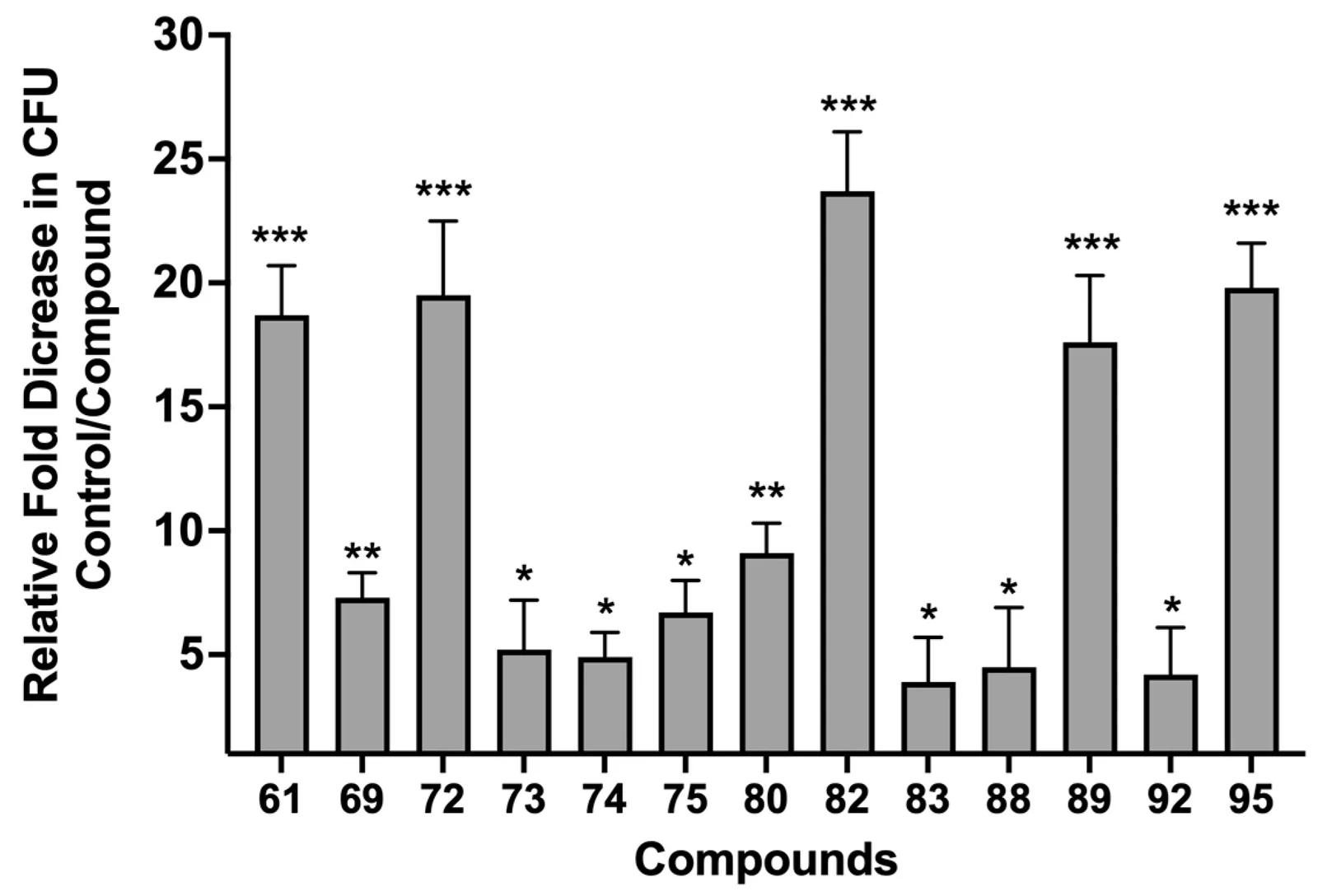
Validation of trehalose transporter-specific inhibitors by H_2_O_2_ susceptibility assay. Mtb was exposed to oxidative stress (0.4% H_2_O_2_ for 1 h) in the presence or absence of test compounds at subinhibitory concentrations under carbon-limiting conditions (0.02% glucose). Survival following H_2_O_2_ treatment was assessed by serial spot dilution and CFU enumeration. Results are presented as the relative fold decrease in CFU for compound-treated Mtb relative to untreated controls, providing a quantitative measure of transporter-dependent oxidative-stress sensitivity. Data are presented as the mean ± SD of three independent biological experiments (n = 3), each performed in eight technical replicates. Statistical comparisons between compound-treated and untreated Mtb were performed using one-way ANOVA with Dunnett’s post hoc multiple-comparison test against the untreated control; * *p* < 0.05, ** *p* < 0.01, and *** *p* < 0.001.

After establishing the baseline, we used the H_2_O_2_ susceptibility assay to identify compounds that functionally disturb the LpqY-SugABC transport system in Mtb. All 23 compounds were evaluated at optimized concentrations under carbon-limiting conditions and subjected to H_2_O_2_ phenotypic screening. Among the 23 compounds, the thiourea-containing compounds, **61**, **72**, **82**, and **95**, exhibited the strongest inhibition of Mtb growth, reducing the wild-type survival to levels comparable to those of the LpqY-SugABC^(–)^ mutant, suggesting potent inhibition of the transporter (Figure 5). An additional nine compounds (**69**, **73**–**75**, **80**, **83**, **88**, **89**, and **92**) produced moderate reductions in H_2_O_2_ resistance relative to the wild-type controls. The remaining ten compounds had no detectable effect on survival under the tested conditions.

### 2.7. Metabolic Labeling of Mtb via the LpqY-SugABC Transporter In Vitro and In Situ

To confirm the specificity of FITC-tre incorporation through the LpqY-SugABC transporter and validate it as a functional target for metabolic labeling, we examined FITC-tre uptake in Mtb wild-type, LpqY-SugABC^(−)^ knockout, and LpqY-SugABC^(+)^-complemented strains in vitro and in culture macrophages. All strains constitutively expressed tomato red fluorescent protein to enable simultaneous bacterial visualization.

**Figure 6:**
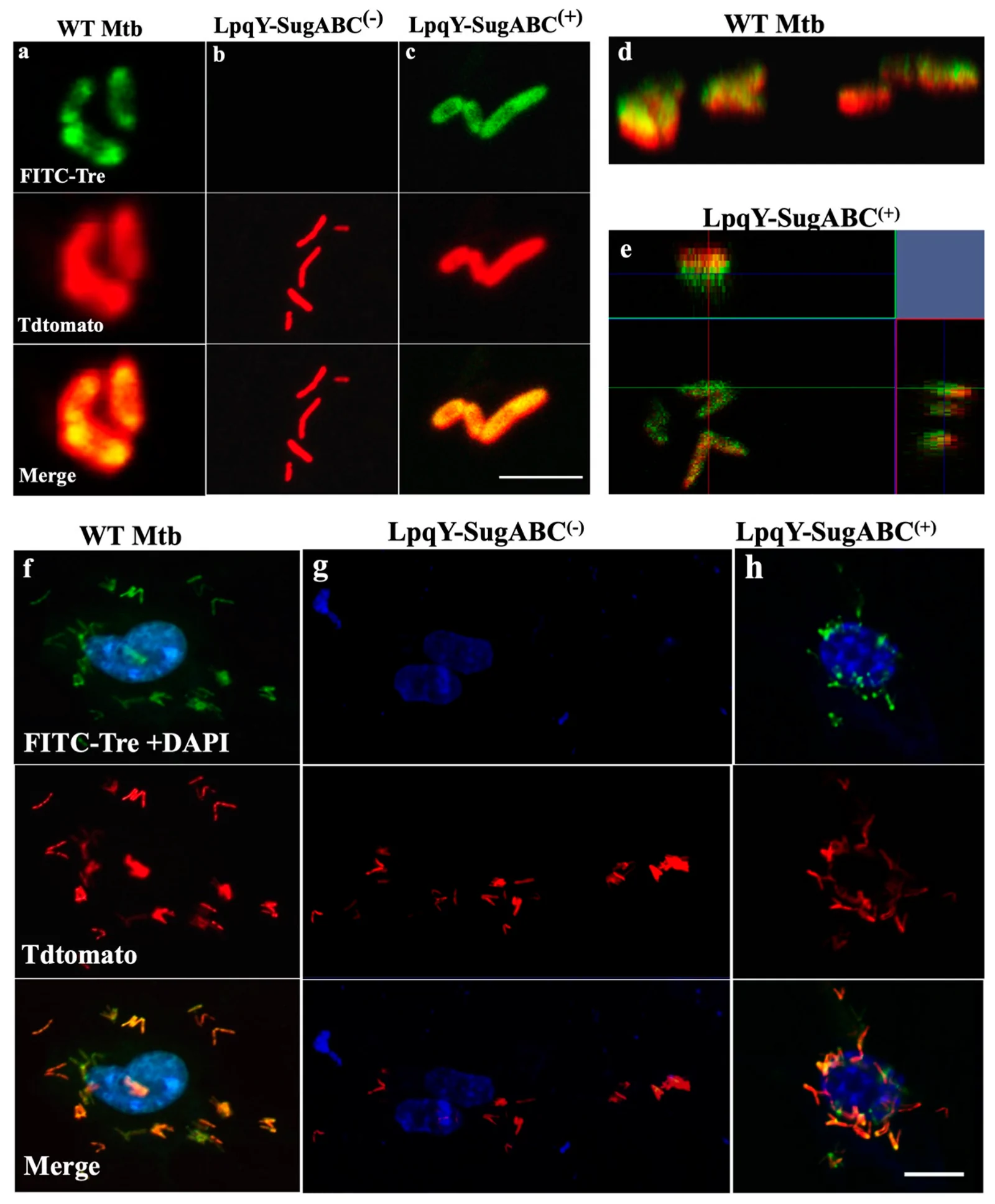
Metabolic labeling of Mtb with FITC-tre in vitro and within the host macrophages. (**a**–**c**) *In vitro* FITC-tre labeling of the Tdtomato protein expressing Mtb wild-type, LpqY-SugABC^(−)^ knockout, and LpqY_SugABC^(+)^-complemented clones. Scale bar: 2 μm. (**d**,**e**) The z section of FITC-Tre labeled wild type and LpqY_SugABC^(+)^ Mtb reveals three-dimensional distribution of FITC-tre at the bacterial cell wall. (**f**–**h**) Confocal micrographs of infected THP-1 macrophages (MOI 10, 24 h FITC-tre exposure) demonstrate the colocalization of FITC-tre with Mtb wild type and complemented clone, whereas (**g**) LpqY-SugABC knockout clone failed to incorporate the probe within infected macrophages. Nuclei are stained with DAPI (blue). Scale bar: 5 μm.

When cultured in vitro with FITC-tre (100 μM), Mtb wild type and the LpqY-SugABC^(+)^-complemented strain displayed robust and consistent FITC labeling, with fluorescent signal predominantly localized to the bacterial cell wall and poles (Figure 6a,c). In contrast, the LpqY-SugABC^(−)^ knockout strain cultured under identical conditions failed to accumulate significant FITC-tre signal above background levels (Figure 6b). Quantitative analysis revealed that mean fluorescence intensity (MFI) of FITC signal per bacterial cell was significantly higher in the wild-type (MFI = 487 ± 89 arbitrary units [AU]) and complemented (MFI = 512 ± 76 AU) strains compared to the knockout strain (MFI = 68 ± 31 AU; *p* < 0.001, ANOVA). The percentage of bacteria displaying significant FITC labeling was 89 ± 6% for the wild type and 91 ± 5% for complemented strains, whereas only 8 ± 3% of knockout bacteria exceeded the labeling threshold. Z-section analysis of FITC-labeled wild type and the LpqY-SugABC^(+)^ Mtb confirmed that FITC-tre signal accumulated specifically at the bacterial cell wall and membrane, with three-dimensional reconstruction revealing concentrated labeling at bacterial poles and along the cell-wall perimeter (Figure 6d,e). This subcellular localization is consistent with the known distribution of the LpqY-SugABC transporter complex at the mycobacterial cell surface. These results demonstrate that FITC-tre uptake is dependent on functional LpqY-SugABC transporter activity.

Furthermore, to assess the functional capacity of the LpqY-SugABC transporter to mediate trehalose uptake during intracellular infection, we evaluated FITC-tre labeling of Mtb growing within human THP-1 macrophages. Following infection at an MOI of 10 for 2 h, macrophages were supplied with FITC-tre and incubated for an additional 24 h to permit intracellular bacterial growth and metabolic labeling. Confocal microscopy of infected THP-1 cells revealed robust FITC-tre signal accumulation in Mtb wild type and LpqY-SugABC^(+)^ residing within macrophages by 24 h post-infection. (Figure 6f,h). Colocalization analysis confirmed spatial overlap between FITC-tre (green) and tomato red-expressing bacteria (red), resulting in a yellow merged signal that indicated FITC-tre incorporation into intracellular Mtb. In contrast, LpqY-SugABC^(−)^ knockout bacteria within infected macrophages failed to incorporate FITC-tre and remained predominantly red (Figure 6g), demonstrating absence of probe uptake despite exposure to the same intracellular environment as the wild-type and complemented strains. Quantitative analysis of intracellular labeling revealed that the mean percentage of Mtb wild type colocalized with FITC-tre was 84 ± 7%, comparable to the complemented strain (87 ± 6%), while only 9 ± 4% of knockout bacteria showed significant FITC-tre colocalization (*p* < 0.001, ANOVA). Manders’ overlap coefficients confirmed significantly greater colocalization in the wild-type (0.82 ± 0.08) and complemented (0.85 ± 0.06) strains compared to knockout bacteria (0.12 ± 0.05; *p* < 0.001) (Figure 6f–h). These findings demonstrate that the LpqY-SugABC transporter functions as the primary mechanism for trehalose acquisition in intracellular Mtb and that its activity is preserved within the macrophage cytoplasm, indicating that this system remains metabolically active during host-cell infection.

### 2.8. Assessing Compound Specificity Against LpqY-SugABC Transporter Using the FITC-Tre Incorporation Assay

**Figure 7:**
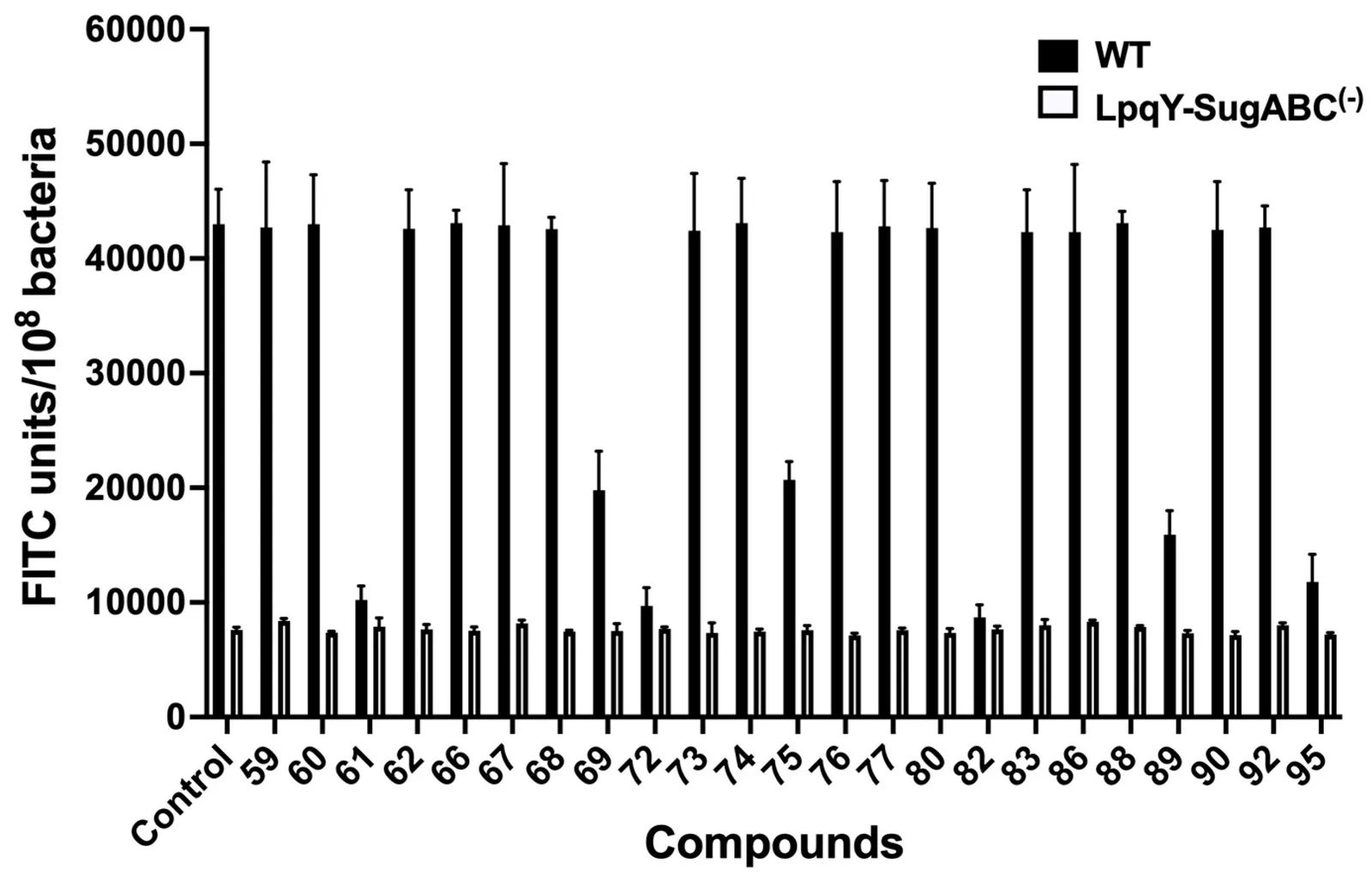
The effects of compounds on Mtb cell-wall labeling with FITC-tre. The wild-type and LpqY-SugABC^(−)^ Mtb strains were cultured in Middlebrook 7H9 medium at an initial O.D. of 0.5 and treated with test compounds at their respective IC_50_ concentrations for 3 h before the addition of 100 μM FITC-Tre. Cultures supplemented with FITC-Tre without compound treatment served as controls. After 24 h, cells were harvested and washed, and fluorescence was measured using a microplate fluorometer (excitation, 485 nm; emission, 525 nm). Bars represent fluorescence intensity (FITC units per 10^8^ bacteria) for the wild-type (black) and LpqY-SugABC^(−)^ (white) strains. Data are presented as the mean ± SD of three independent biological experiments (n = 3), each performed in four technical replicates.

As an alternative to the H_2_O_2_ assay for confirming and establishing compounds that specifically inhibit the LpqY-SugABC trehalose transporter, we compared FITC–tre accumulation in the wild-type and LpqY-SugABC^(−)^ knockout Mtb strains (Figure 7). The Mtb wild type accumulated high levels of FITC-tre (45,000 ± 5200 FITC units/10^8^ bacteria) through functional transporter-mediated uptake, whereas the knockout mutant showed only minimal accumulation (8100 ± 1200 FITC units/10^8^ bacteria), establishing a 5.6-fold difference that provided a robust baseline for detecting transporter-specific inhibitors. All 23 compounds identified in the initial phenotypic screen were evaluated using this assay. Based on their effects on FITC-tre uptake, the compounds segregated into two activity groups. Seven compounds significantly reduced FITC-tre accumulation in the wild-type strain. Among these, the strongest inhibitors were thiourea-containing compounds **61**, **72**, and **82** decreased FITC-tre uptake to levels comparable to the knockout strain (7000–12,200 FITC units/10^8^ bacteria), consistent with near-complete inhibition of transporter activity. Compounds **69**, **75**, **89**, and **95** produced a more moderate reduction (50–90%), decreasing fluorescence to 16,200–20,100 FITC units/10^8^ bacteria, indicative of partial transporter inhibition. In contrast, the remaining 16 compounds did not significantly reduce FITC-tre accumulation in the wild type, suggesting that their activity potentially could be mediated through targets downstream of trehalose transport rather than inhibition of the LpqY-SugABC transporter. Critically, the LpqY-SugABC^(−)^ knockout strain remained unaffected by all compound treatments, maintaining baseline fluorescence of 8100 ± 1200 FITC units/10^8^ bacteria regardless of whether compounds were present, providing evidence that the identified inhibitors specifically target the transporter and do not exert general cytotoxic effects.

### 2.9. Trehalase Inhibition Assay

**Figure 8:**
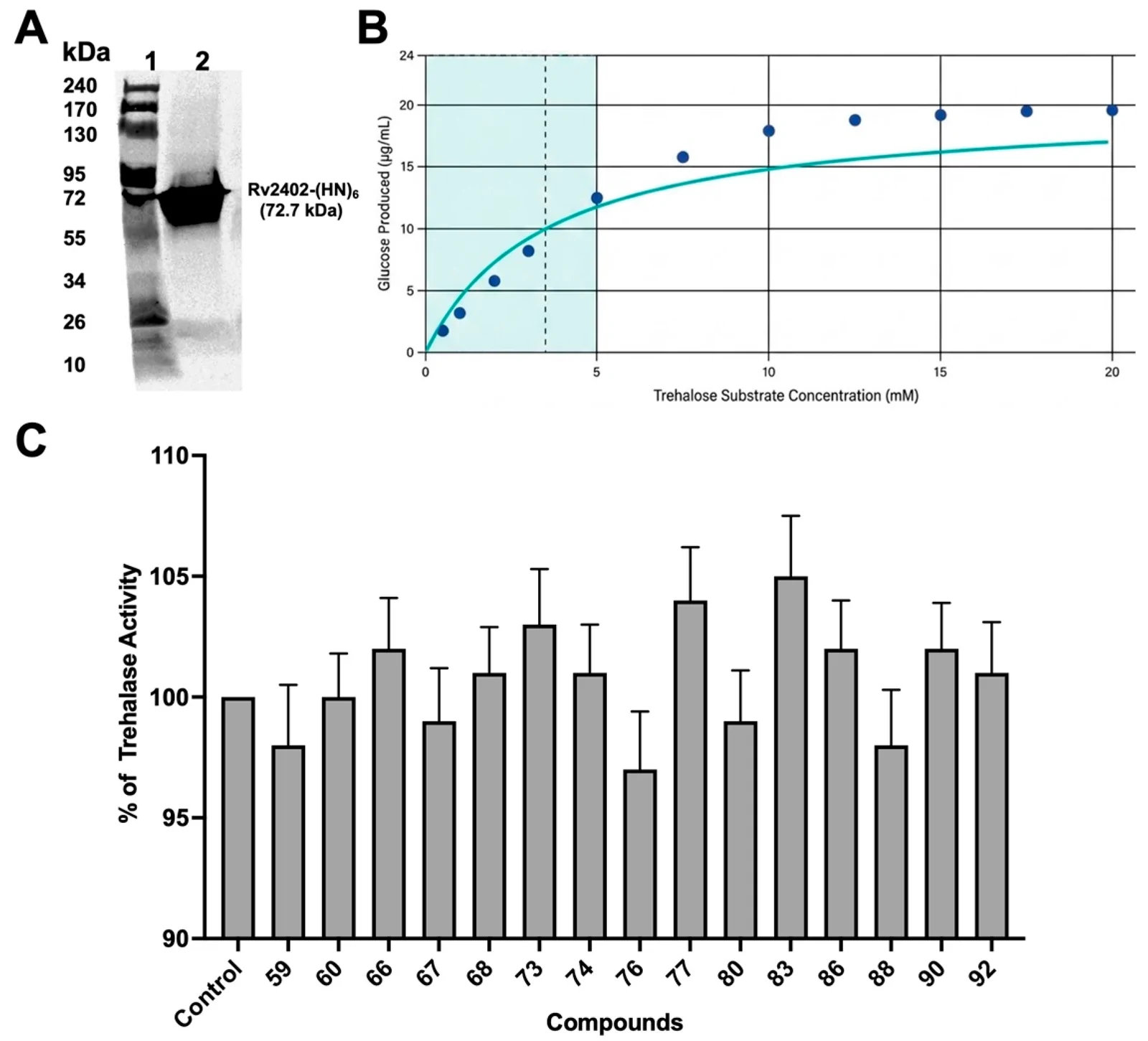
Purification and biochemical characterization of recombinant trehalase. (**A**) SDS-PAGE analysis of the purified recombinant trehalase. Lane 1, molecular weight marker (kDa); and Lane 2, purified recombinant trehalase, (~72 kDa). (**B**) Michaelis–Menten kinetics of trehalase activity. Glucose produced (µg/mL) is plotted as a function of trehalose substrate concentration (mM). Data points represent measured glucose production at each substrate concentration, and the curve represents the nonlinear regression fit used to determine the kinetic parameters. The shaded region (0–5 mM) highlights the substrate concentration range used for initial velocity determination, with the dashed line indicating the estimated Km value. (**C**) Screening of candidate compounds for inhibitory or modulatory effects on trehalase activity. Enzymatic activity in the presence of each compound is expressed as a percentage of the untreated control (set to 100%). Bars represent mean ± SD of trehalase activity for each compound tested relative to control, from technical triplicates across two independent experiments (n = 2). Compound-treated groups were compared to the vehicle-only control using one-way ANOVA with Dunnett’s post hoc multiple-comparison test. No comparisons reached statistical significance (*p* > 0.05 for all compounds tested).

To determine whether the phenotypic anti-TB activity of the screening hits was mediated through inhibition of downstream trehalose-metabolizing enzymes, we expressed and purified recombinant Mtb Rv2402 trehalase (Figure 8A) and evaluated its sensitivity to compound inhibition. We focused on 16 of the 23 compounds, excluding the seven compounds whose FITC-tre incorporation and H_2_O_2_ susceptibility assays provided complementary evidence of specific LpqY-SugABC transporter inhibition. The purified Rv2402 enzyme exhibited strong baseline trehalase activity under the optimized assay conditions (Figure 8B). Enzymatic reactions performed at 37 °C using 50 µg of purified enzyme demonstrated consistent hydrolysis of trehalose across the tested substrate concentration range of 0–20 mM, confirming that the recombinant enzyme was catalytically active.

For subsequent inhibition studies, a substrate concentration of 5 mM trehalose was selected because it provided a strong and reproducible assay signal while maintaining sufficient sensitivity to detect decreases in enzymatic activity. Under these conditions, the reaction generated approximately 12.5 µg/mL glucose, representing an optimal balance between signal intensity and dynamic range. Compounds were then tested at subinhibitory concentrations using a colorimetric glucose-based assay. Remarkably, none of the tested compounds produced detectable inhibition of Rv2402 trehalase activity, as measured by quantifying the glucose concentration generated from trehalose conversion (Figure 8C). Trehalose was efficiently cleaved to glucose across all compound treatments, with enzyme activity remaining within 95–105% of the vehicle-only control.

## 3. Discussion

Antibiotic-treatment failure in tuberculosis (TB) is conventionally attributed to genetic drug resistance; however, a growing body of evidence indicates that phenotypic drug tolerance in genetically susceptible *M. tuberculosis* populations is an equally important, and far less understood, contributor to prolonged therapy and relapse [5,6,18]. Tolerant subpopulations survive drug pressure long enough to permit the emergence and selection of genetically resistant strains [5,6], while the prolonged chemotherapy regimens required for treatment further amplify this risk by placing sustained demands on patient adherence over an extended therapeutic window [4]. Although several new agents have entered the anti-TB pipeline in recent years, most have been developed largely empirically, and combination regimens are still assembled primarily on the basis of clinical experience rather than a rational, mechanism-based strategy [32,33]; in some cases, this has led to unanticipated antagonism between co-administered drugs [34].

Our group has previously shown that Mtb survives lethal exposure to isoniazid, rifampicin, moxifloxacin, mefloquine, and bedaquiline not by dying immediately, but through a coordinated proteomic remodeling response involving metabolic enzymes and transport systems that sustain viability for six or more days under otherwise bactericidal conditions [14]. Our study identified a discrete set of “escape” proteins shared across multiple mechanistically unrelated drug classes [14], and functional disruption of several of these targets, most notably the trehalose-specific transporter LpqY-SugABC, synergistically accelerated Mtb killing by isoniazid, rifampicin, and bedaquiline both in axenic culture and within infected human macrophages [17]. The present study builds directly on that observation, moving from genetic proof of concept to small-molecule discovery, with the goal of identifying compounds that pharmacologically phenocopy the LpqY-SugABC knockout and could therefore be developed as adjunctive, tolerance-breaking agents for existing anti-TB regimens.

Rather than screening against purified LpqY-SugABC protein, we designed a phenotypic screen anchored in bacterial physiology rather than a single molecular target, building on our group’s established track record of identifying small-molecule inhibitors of mycobacteria through phenotypic approaches [35]. We developed the carbon source-restricted phenotypic assay that forces mycobacteria to survive and depend on trehalose uptake and recycling for viability. This design reflects the metabolic flexibility Mtb requires to survive in the nutrient-poor host environment and the physiologically relevant condition under which trehalose recycling becomes essential for cell-wall maintenance and energy metabolism [11,21]. This carbon source-restricted activity profile, growth inhibition specifically under trehalose-dependent conditions with no detectable effect in glucose-supplemented medium or nutrient-rich 7H9 broth (Figure 1), was the defining selection criterion for this compound set and reflects the intended mechanism of action of these hits rather than a limitation of their antibacterial potential. Because Mtb can freely utilize glucose and other abundant carbon sources when available, the trehalose recycling pathway is metabolically dispensable under glucose-replete or rich-broth conditions; consequently, compounds acting through this pathway are not expected to show activity under standard nutrient-rich growth conditions, including those used in conventional CLSI-based susceptibility testing. Instead, these compounds selectively target the physiological state in which Mtb depends on trehalose salvage for survival, a condition believed to more closely approximate the nutrient-restricted intracellular macrophage environment encountered during infection. Accordingly, potency for this compound class was assessed as IC50 under trehalose-restricted minimal medium conditions (Table 2) rather than as a conventional MIC in rich medium and further evaluated against intracellular Mtb within infected macrophages, a physiologically intact host-cell system in which the relevant carbon-limited condition is naturally imposed.

Assay performance and hit-selection criteria followed established HTS design and validation principles for anti-tubercular compound screening [36], in line with recent large-scale phenotypic screening platforms developed for physiologically relevant, macrophage-based Mtb assays [30]. The screen yielded 23 confirmed hits in Mtb spanning several chemically distinct scaffolds, including propanamides, thioureas, benzamides, and carboxamides, a level of chemical diversity consistent with what is typically recovered from fragment- and phenotype-based screening campaigns against Mtb [37,38]. Several of these chemotypes have previously been reported to act on other validated Mtb targets, including cytochrome P450 CYP125A1, oleic acid catabolism, tryptophan synthase, and MmpL3 [38], raising the possibility that a subset of our hits acted through these previously described mechanisms rather than through trehalose-pathway components specifically. This is an expected and, in our view, informative outcome of a physiology-based phenotypic screen: it necessitates orthogonal target-deconvolution assays to separate transporter-directed compounds from those with unrelated or polypharmacological mechanisms, precisely the rationale for the multi-tiered validation cascade we subsequently applied.

To build the strain toolkit required for target deconvolution, we used an Mtb LpqY transposon mutant and generated a matched LpqY-SugABC^(+)^-complemented strain, following the same integrative complementation strategy previously validated by our group using the pMV306 vector system for functional restoration of virulence-associated loci [17]. RT-PCR confirmed that transposon insertion within *lpqY* exerted a strong polar effect, silencing the entire downstream *sugA*-*sugB*-*sugC* genes, while transcription of the upstream *Rv1234* gene in the operon was unaffected, consistent with our earlier characterization of this genetic locus [17]. This organization is consistent with the operon structure and essentiality profile originally described for LpqY-SugABC by Kalscheuer et al., who established this ABC transporter as the sole route of trehalose recycling and a determinant of in vivo virulence [17]. Complementation fully restored operon-wide transcription, generating an isogenic strain set of the wild type, knockout, and complemented that provided the genetic backbone for every subsequent validation assay in this study. Using this system, we confirmed that loss of LpqY-SugABC function reproduces the accelerated, tolerance-breaking killing phenotype we originally described for INH, RMP, and TMC [14], while susceptibility to MXF remained essentially unchanged regardless of transporter status. This drug-class selectivity is consistent with our earlier finding that the trehalose-recycling escape pathway is not uniformly engaged by all anti-TB drug mechanisms, and it parallels reports that fluoroquinolone activity against Mtb follows distinct pharmacodynamic kinetics from cell-wall- and respiration-targeting agents [39].

Our results demonstrate that compound-mediated enhancement of rifampicin activity largely, but not completely, tracks with transporter inhibition. Consistent with our earlier genetic data [14], several of the trehalose-pathway hits markedly potentiated RMP-mediated killing of intracellular Mtb, in some cases reducing bacterial burden below the level achieved by RMP or INH monotherapy alone. This is notable given that immune-activated macrophages are known to independently induce drug-tolerant phenotypes in Mtb [18], suggesting that transporter inhibitors may act by removing one of the specific metabolic buffers the pathogen relies on within this hostile intracellular niche. Cross-referencing the synergistic compounds against the two independent, transporter-specific assays used in this study, H_2_O_2_ hypersensitization and FITC-tre uptake inhibition, showed good overall concordance for compounds **61**, **69**, **72**, **75**, **82**, **89**, and **95**, supporting a shared mechanism of action centered on trehalose transport inhibition. Among the eight compounds that synergized with RMP in macrophages, however, the strength of orthogonal support was not uniform. Compounds **72** and **82** fell within this concordant, strong-evidence group, whereas compounds **73**, **80**, **83**, and **88** showed only a moderate reduction in H_2_O_2_ resistance and no significant inhibition of FITC-tre uptake, placing them in an intermediate category: consistent with some degree of transporter engagement, but without the convergent, high-confidence signal seen for the strongest hits. Two synergistic compounds, **77** and **90**, did not phenocopy the knockout in either orthogonal assay. This suggests that not every compound scoring in the primary carbon source-restricted screen, nor every compound synergizing with rifampicin in macrophages, necessarily acts through LpqY-SugABC itself; the intermediate compounds (**73**, **80**, **83**, and **88**) may reflect partial or lower-affinity transporter inhibition, activity on a related but distinct target within the same pathway, or a mixed mechanism, while compounds **77** and **90** may instead act downstream in cell-envelope biogenesis or through an independent mechanism that nonetheless intersects functionally with the same tolerance pathway. Given that trehalose-pathway disruption and general cell-wall stress can produce overlapping physiological consequences, we cannot fully exclude an indirect, permeabilization-based contribution for these two compounds, and we consider the intermediate group (**73**, **80**, **83**, and **88**) to be moderate-priority, and **77** and **90** to be lower-priority, candidates for LpqY-SugABC-specific mechanism-of-action studies pending further characterization.

This study extends prior findings by showing that trehalose recycling is linked to oxidative stress defense. A key mechanistic extension of the present work beyond our earlier proteomic and genetic study [14] is the demonstration that loss of trehalose recycling sensitizes both *M. smegmatis* and Mtb to hydrogen peroxide under carbon-limiting conditions, and that this phenotype is reversed by genetic complementation. We recognize that increased H_2_O_2_ susceptibility is not, by itself, a specific phenotype, as diverse metabolic and cell-envelope defects can independently increase oxidative stress sensitivity. Our H_2_O_2_ susceptibility assay was based on the findings of Pohane et al. [23], demonstrating that trehalose recycling supports energy-efficient cell-envelope biosynthesis and that disruption of this pathway compromises envelope integrity. We therefore hypothesized that loss of LpqY-SugABC-mediated trehalose recycling would similarly increase oxidative susceptibility. Consistent with this prediction, targeted deletion of *sugC* and *lpqY* in *M. smegmatis* and transposon disruption of *lpqY* in Mtb each increased H_2_O_2_ susceptibility, with resistance restored by genetic complementation in Mtb (Table 2). This oxidative hypersensitivity is best interpreted as a downstream physiological consequence of losing trehalose recycling, plausibly via compromised envelope integrity, rather than as direct evidence that a compound inhibits the transporter itself. We therefore used H_2_O_2_ hypersensitization as a functional correlate of transporter loss, not a proof of physical target engagement. Because trehalose and its glycolipid derivatives, TDM and TMM, are structural components of the mycobacterial outer envelope that also modulate host immune engagement [17,20], and because trehalose recycling itself has been shown to support energy-efficient biosynthesis of this envelope [23], a defect in trehalose salvage likely compromises envelope integrity in a manner that increases vulnerability to reactive oxygen species generated within the phagosome [40]. Accordingly, compounds that sensitize Mtb to H_2_O_2_ are best described as reproducing this downstream consequence of transporter loss, and their nomination as candidate transporter inhibitors depends on convergence with the FITC-tre uptake assay described below. This finding provides a plausible physiological explanation for why LpqY-SugABC disruption specifically potentiates killing by drugs such as isoniazid and bedaquiline, both of which are known to generate secondary oxidative stress as part of their bactericidal mechanism [41], while leaving MXF susceptibility largely unaffected. Beyond its use here as a mechanistic readout, the H_2_O_2_ hypersensitivity assay proved to be a robust, orthogonal counter-screen for nominating compounds, **61**, **72**, **82**, **89**, and **95**, as strong candidate transporter inhibitors, independent of the FITC-tre uptake platform.

Furthermore, our results demonstrate that FITC-tre labeling visualizes transporter-dependent uptake and compound inhibition. Using the same fluorescent trehalose probe strategy previously applied to demonstrate LpqY-SugABC-dependent labeling of Mtb in vitro and within infected macrophages [28], we confirmed that probe incorporation is essentially abolished in the LpqY-SugABC^(−)^ knockout and fully restored upon complementation, both in culture and in the intracellular macrophage environment. The original design and validation of unnatural trehalose analogs as chemical reporters of Mtb trehalose metabolism, established by Backus et al. [28], underlies the specificity of this readout and supports its use here as a quantitative, transporter-dependent uptake assay rather than a general marker of bacterial viability. This result reinforces that LpqY-SugABC-mediated uptake remains active and accessible during intracellular infection. Because LpqY is annotated as a lipoprotein anchored to the outer face of the plasma membrane, this uptake activity is consistent with the proposed surface accessibility of the transporter; however, our probe data demonstrate functional, uptake-level accessibility rather than directly resolving the topology of transporter or confirming that inhibitor binding occurs extracellularly. Applying this assay to our compound set as a quantitative readout of transporter function identified thiourea-containing compounds as the most potent inhibitors of FITC-tre uptake, closely mirroring the compounds independently flagged by the H_2_O_2_ assay. The convergence of two mechanistically distinct, orthogonal assays on a common subset of hits substantially strengthens confidence that these compounds act specifically at the level of the LpqY-SugABC transporter rather than through nonspecific toxicity or off-target effects.

In addition, because trehalose imported via LpqY-SugABC is ultimately hydrolyzed intracellularly to glucose by trehalase (TreA/Rv2402) before entering central carbon metabolism [42], we tested whether any of our hits acted at this downstream enzymatic step rather than at the transporter itself. For this purpose, recombinant Rv2402 was expressed with a 6 × HN affinity tag fused to the C-terminus, leaving the native N-terminus unmodified. Mtb Rv2402 trehalase belongs to the GH15 family, whose catalytic site is located within an (α/α)_6_-barrel domain, away from the C-terminus, making interference by a short C-terminal tag unlikely. Consistent with this structural organization, the purified enzyme displayed robust, substrate-saturable kinetics over the tested trehalose concentration range (Figure 8B), indicating retained catalytic function under the assay conditions used. None of tested compounds produced detectable inhibition of recombinant Rv2402 activity, despite robust baseline enzymatic activity under the assay conditions used. Although a comparison with untagged, native Rv2402 was not performed and a modest quantitative effect of the C-terminal tag on catalytic efficiency cannot be completely excluded, the purpose of this assay was to assess compound-mediated inhibition of trehalose hydrolysis rather than to define precise native kinetic parameters. Accordingly, the C-terminally tagged construct was considered suitable for this comparative analysis. Our results rule out TreA-mediated trehalose hydrolysis as the mechanism of action for our hits, but it does not identify the precise binding site of these compounds within the transport process, nor does it exclude effects on other downstream metabolic steps not tested here. Taken together with the transporter-uptake and oxidative-stress data, this result helps localize the mechanism of action of our lead compounds to the transport step of trehalose recycling rather than to intracellular trehalose catabolism and argues against a generalized inhibitory effect on trehalose-processing enzymes as a class. This distinction is potentially important from a drug-development standpoint since, as the LpqY-SugABC is a periplasmic/surface-exposed uptake system, transporter-directed inhibitors may retain the permeability advantage originally proposed for this target [17], whereas TreA, being cytoplasmic, would not offer the same accessibility benefit.

Several limitations should be considered when interpreting these findings. First, the primary screen was conducted in *M. smegmatis* as a fast-growing surrogate for Mtb; although most hits were confirmed in Mtb CDC1551, phenotypic differences between the two species in trehalose metabolism cannot be fully excluded. Second, compounds were screened at a single concentration in the primary assay, which may have led to under-representation of weakly active but chemically tractable series, a known limitation of single-point HTS designs [31]. Third, and most importantly, the evidence presented here for LpqY-SugABC as the mechanistic target of our lead compounds remains indirect. The FITC-tre uptake and H_2_O_2_ hypersensitization assays demonstrate that compound treatment functionally phenocopies genetic loss of the transporter, and their convergence on a common subset of hits (**61**, **72**, and **82** in particular) strengthens confidence in a shared, transporter pathway-dependent mechanism. However, neither assay demonstrates direct physical interaction between these compounds and the LpqY-SugABC complex, nor do they define a binding site, binding affinity, or stoichiometry of interaction. Establishing direct target engagement would require complementary biophysical approaches, such as surface plasmon resonance, isothermal titration calorimetry, or thermal shift assays using purified, reconstituted transporter, together with molecular docking supported by site-directed mutagenesis of predicted ligand-binding residues. These experiments were not performed in the present study, in part because functional reconstitution of this multi-subunit membrane transporter for biophysical binding studies presents a substantial technical undertaking beyond the scope of this phenotypic discovery effort. Consequently, our conclusions should be interpreted as identifying compounds that act through a trehalose transport-dependent mechanism based on convergent functional evidence, rather than as establishing physical target engagement; biophysical validation of direct binding represents an important priority for future characterization of our lead compounds. Furthermore, structural overlap between several of our hit chemotypes and previously reported inhibitors of CYP125A1, MmpL3, and tryptophan synthase [38] means that target promiscuity remains a formal possibility for compounds not yet subjected to resistant-mutant selection or biophysical target-engagement studies; this is particularly relevant for compounds **77** and **90**, whose activity was not corroborated by either the H_2_O_2_ or FITC-tre assays. Finally, while our data establish potent in vitro and intracellular macrophage activity, in vivo efficacy, pharmacokinetics, and combination dosing with front-line anti-TB drugs remain to be evaluated, and persistence-related phenotypes observed in cell culture do not always fully predict in vivo behavior [4,18].

The findings of this study extend our earlier proteomic and genetic identification of LpqY-SugABC as an “escape” pathway target that prolongs Mtb survival during antibiotic exposure [14] by providing the first evidence that this system is druggable with small molecules identified through unbiased phenotypic screening. The convergence of orthogonal transporter-function assays on a core set of thiourea- and propanamide-containing hits, most notably compounds **61**, **72**, and **82**, nominates these as priority candidates for medicinal chemistry optimization and in vivo efficacy testing. More broadly, this work supports the concept originally proposed in our earlier study [14], that rationally pairing primary anti-TB drugs with inhibitors of the metabolic escape pathways that they themselves induce may accelerate bacterial killing, shorten the duration of effective therapy, and reduce the window during which drug-tolerant subpopulations can evolve into genetically resistant strains [14,18].

## 4. Materials and Methods

### 4.1. Chemicals and Compound Libraries

The Maybridge HitFinder collection (14,400 compounds) and the ChemBridge DIVERSet small-molecule library (35,600 compounds) were obtained from the Oregon State University College of Pharmacy High-Throughput Screening Services Laboratory and screened at a single concentration of 10 μM in 1% DMSO. Antibiotics, rifampicin and isoniazid, were purchased from Sigma-Aldrich (St. Louis, MO, USA). The phorbol 12-myristate 13-acetate (PMA) and resazurin were also from Sigma-Aldrich. The fluorescent small-molecule probe FITC-trehalose (FITC-tre) was purchased from Kerafast (Boston, MA, USA).

### 4.2. Bacterial Strains

*Mycobacterium smegmatis* mc^2^155 (ATCC 700084) was obtained from the American Type Culture Collection (ATCC). *M. tuberculosis* CDC1551 (NR-13649; designated as Mtb wild type) and *M. tuberculosis* CDC1551 transposon mutant 3247 (NR-18886; designated as LpqY-SugABC^(−)^) were obtained from the Biodefense and Emerging Infections (BEI) Research Resources Repository. *M. smegmatis* mc^2^155 Δ*sug*C and Δ*lpq*Y gene knockout mutants were kindly provided by Dr. W.R. Jacobs Jr. at the Albert Einstein College of Medicine, New York. Mycobacteria were cultured on Middlebrook 7H10 agar or in Middlebrook 7H9 broth (Difco Laboratories, Detroit, MI, USA) supplemented with 10% oleic acid–albumin–dextrose–catalase (OADC; Hardy Diagnostics, Santa Maria, CA, USA), 0.05% Tween-80, and 0.5% glycerol (7H9 T20). Strains were passaged on 7H10 agar for fewer than 15 passages. To prepare bacterial suspensions, mid log-phase cultures grown on 7H10 agar were scraped and resuspended in 10 mL minimal medium or Hanks’ Balanced Salt Solution (HBSS; VWR, West Chester, PA, USA). Cells were sonicated in a water bath for 1 min, passed through a 22-gauge syringe 10 times, and allowed to settle for 15 min. The upper 9 mL supernatant was transferred to a new tube, and the optical density at 600 nm was measured. Cells were then diluted to the desired concentration using the conversion factor 1.0 OD_600_ = 3 × 10^8^ cells/mL. Minimal medium (MM) was prepared as previously described [43].

### 4.3. Tissue Culture Cell Lines

The human lung carcinoma cell line A549 (ATCC CCL-185) was used to assess compound-mediated cytotoxicity and cultured in Dulbecco’s Modified Eagle’s Medium (DMEM) supplemented with 10% heat-inactivated fetal bovine serum (FBS). Human monocytic THP-1 cells (ATCC TIB-202) were used to assess compound-mediated cytotoxicity and intracellular bacterial survival. THP-1 cells were cultured in RPMI 1640 medium (Cellgro, Manassas, VA, USA) supplemented with 10% FBS, 2 mM L-glutamine, and 25 mM HEPES (RP10), and maintained at 37 °C in a humidified atmosphere containing 5% CO_2_. Cultures were maintained at densities below 1 × 10^6^ cells/mL and used for fewer than 10 passages. Prior to infection, THP-1 cells were differentiated into macrophage-like cells. Briefly, cells were seeded at 5 × 10^5^ cells per well in clear, flat-bottom 96-well plates containing RPMI medium and treated overnight with 50 ng/mL PMA. The following day, the medium was replaced with 200 μL of fresh RP10 media per well, and cells were rested for an additional 48 h at 37 °C in 5% CO_2_ before cytotoxicity or infection assays.

### 4.4. High-Throughput Screening (HTS) Assay

Compounds were prepared as DMSO stock solutions and transferred by automated liquid handling into clear, flat-bottom 384-well assay plates. Compounds were screened at a final concentration of 10 µM with a final DMSO concentration of 1% (*v*/*v*). To prepare bacterial inoculum, *M. smegmatis* mc^2^155 grown to mid-log phase on 7H10 plates was heavily inoculated into 10 mL MM containing 20 mM glucose in a 50 mL conical tube and incubated overnight at 37 °C with shaking. On the next day, cells were pelleted (5000× *g*, 15 min), the supernatant was removed, and the pellet washed three times in 10 mL MM lacking a carbon source. A single-cell suspension was prepared as described above and diluted in MM to an OD_600_ of 0.03–0.05. Each well of the 384-well plate already contained 25 µL MM with compound (final compound concentration 10 µM in 1% DMSO). Then, 25 µL of *M. smegmatis* inoculum and 50 µL of 20 mM trehalose solution in MM were added (for a final concentration of 10 mM). For the primary screen, the MM contained only trehalose as a carbon source. Negative control wells contained bacteria in MM without trehalose (no-carbon control). Growth control wells contained bacterial culture in 7H9 broth (rich media). Plates were sealed, placed in plastic bags to minimize evaporation, and incubated statically at 37 °C for 72–96 h. Then, 5 µL of resazurin (5 mg/mL) was added to each well, and the plates were incubated statically at 37 °C until a color/fluorescence change occurred (4–6 h). Growth inhibition was assessed using two complementary readouts of the resazurin-to-resorufin conversion: fluorescence intensity (excitation 530 nm/emission 590 nm, Tecan plate reader) and colorimetric inspection of the resazurin color change (blue to pink), which reflects the corresponding shift in absorbance maxima between resazurin and resorufin. A compound was scored as a hit if resazurin reduction was inhibited by more than 50% relative to DMSO-treated control wells on the same plate, based on concordant fluorescence and colorimetric readouts. This dual-readout approach provided an internal concordance check across all screening plates. Assay quality was further assessed using the DMSO-treated (maximal growth) and no-carbon-source MM (maximal inhibition) control wells present in six replicate rows (A–F) on every plate (Figure 1, red-boxed columns); fluorescence values from these control populations were used to calculate the Z′-factor and signal-to-background ratio for the primary screen.

### 4.5. Secondary Testing and Triage

Primary hits that inhibited *M. smegmatis* growth in MM supplemented with trehalose were cherry-picked and retested three times in 96-well format across three growth conditions to assess carbon-source specificity and general toxicity: MM containing 10 mM trehalose, MM containing 20 mM glucose, and 7H9 broth (rich medium) as an in vitro growth control. Inoculum density, compound concentrations, and incubation conditions were maintained as described for the primary screen. Compounds that reproducibly inhibited growth in trehalose but not glucose were considered candidate trehalose-pathway inhibitors. The selected compounds were purchased from ChemBridge Corporation and retested in a 96-well assay format using *M. smegmatis*. Compounds with confirmed activity were subsequently evaluated against Mtb.

### 4.6. Compound Activity Validation in M. tuberculosis

The Mtb wild type was grown on 7H10 agar and heavily inoculated into 50 mL conical tubes containing 10 mL MM supplemented with 20 mM glucose. Cultures were incubated overnight at 37 °C with shaking and then centrifuged at 5000× *g* for 15 min. The bacterial pellet was washed twice more with MM lacking a carbon source, and inoculum was prepared by resuspending the bacteria in MM containing either trehalose or glucose at final concentrations of 20 mM and 40 mM, respectively. Suspensions were adjusted to an OD_600_ of 0.03–0.05 as described above. For this screen, clear, flat-bottom 96-well plates were preloaded with 100 µL of selected compounds in MM at a final concentration of 200 µM or DMSO alone as a vehicle control, followed by the addition of 100 µL of Mtb inoculum supplemented with trehalose (10 mM final assay concentration) or glucose (20 mM final assay concentration). In addition, MM without a carbon source and 7H9 broth were inoculated as negative and growth controls, respectively. Plates were sealed, placed in plastic bags, and incubated statically at 37 °C for 7 days. Five microliters of resazurin (5 mg/mL) were then added to each well, and plates were incubated statically at 37 °C until an appropriate color change developed (4–6 h). Fluorescence was measured using a Tecan plate reader at 530 nm excitation and 590 nm emission, and growth inhibition was scored based on the absence of resazurin color conversion.

### 4.7. Cytotoxicity Assay

Hits confirmed in the secondary screen were evaluated for cytotoxicity against differentiated THP-1 macrophages and A549 epithelial cells. Compounds were prepared as 10 mM stock solutions in DMSO and serially diluted in half-log increments to generate the desired concentration range (100–0.01 μM). Cyclosporin A (50 μg/mL) served as a positive control for cytotoxicity, while DMSO alone served as the vehicle control. For compound preparation, 196 μL of RP10 or DMEM medium was dispensed into each well of a 96-well flat-bottom plate, and 4 μL of each compound dilution was added. A549 or differentiated THP-1 cells were prepared as described above. Culture medium was removed and replaced with 95 μL fresh RP10 or DMEM per well, followed by the addition of 95 μL of the diluted compounds using a multichannel pipette. Assays were performed in duplicate. Plates were incubated for 72 h at 37 °C in a humidified atmosphere containing 5% CO_2_. Cell viability was assessed using a resazurin reduction assay. Briefly, 10 μL resazurin solution (50 μg/mL in PBS) was added to each well, and plates were incubated for 3–5 h until an appropriate color change was observed. Fluorescence was measured at excitation and emission wavelengths of 530 and 590 nm, respectively, using a Tecan microplate reader. Cytotoxicity was also evaluated microscopically by examining the integrity of the THP-1 or A549 monolayer. Cell viability was calculated relative to vehicle-treated controls, and the CC50 value was defined as the concentration of compound resulting in a 50% reduction in cell viability.

### 4.8. Intracellular Bacterial Survival Assay

The macrophage monolayers were established by differentiating THP-1 monocytes as described above. The infection was carried out with Mtb wild type at a multiplicity of infection (MOI) of 10. Briefly, bacterial suspensions were prepared in RP10 medium and added to macrophage monolayers for 2 h at 37 °C in a humidified atmosphere containing 5% CO_2_. Following infection, cells were washed three times with HBSS to remove extracellular bacteria and replenished with a fresh RP10 medium containing test compounds at the highest non-toxic concentrations. DMSO-treated wells served as vehicle controls, while isoniazid (2 μg/mL) served as the growth-inhibition control. In addition, for the combination study, selected compounds, at the highest non-toxic concentrations specified in the Section 2, were added together with rifampicin (2 μg/mL). Plates were incubated at 37 °C in 5% CO_2_ for 6 days. Intracellular bacteria were quantified by lysis of host cells with 100 μL of 0.1% Triton X-100. Lysates were serially diluted in HBSS and plated onto Middlebrook 7H10 agar plates. Colonies were enumerated after 14 days of incubation, and bacterial burden was expressed as CFU per well. Data were normalized to the DMSO-treated control and expressed as percent survival relative to untreated infected cells. Statistical analyses were performed using GraphPad Prism (version 11.0). Comparisons between treatment groups were conducted using one-way ANOVA with multiple-comparison testing, or an unpaired Student’s *t*-test where indicated. Statistical significance thresholds are defined in the corresponding figure legends.

### 4.9. Construction of LpqY-SugABC-Complemented Strain

The LpqY-SugABC^(+)^-complemented clone was generated by amplifying the complete 4341-bp LpqY-SugABC transporter genes (*lpqY*, *sugA*, *sugB*, and *sugC*) from *Mycobacterium tuberculosis* CDC1551 genomic DNA using Phusion High-Fidelity DNA Polymerase, with primers flanking the operon and PCR conditions consisting of 35 cycles of 95 °C denaturation (1 min), 63 °C annealing (1 min), and 72 °C extension (4.5 min). The amplified fragment was verified by agarose gel electrophoresis, digested with appropriate restriction enzymes, and ligated into the integrative mycobacterial vector pMV306 carrying an apramycin resistance marker using T4 DNA ligase at a 1:3 vector-to-insert ratio. The recombinant plasmid was first propagated in *Escherichia coli* DH5α, and positive clones were confirmed by restriction analysis before being introduced into the LpqY-SugABC^(−)^ knockout *M. tuberculosis* strain via electroporation (2.5 kV, 25 μF, 1000 Ω). Transformants were recovered and selected on Middlebrook 7H10 agar containing apramycin, and resistant colonies were expanded in liquid medium for further analysis. Successful integration and operon restoration were confirmed by PCR amplification of the full operon from genomic DNA and by RT-PCR analysis of individual genes (*lpqY*, *sugA*, *sugB*, and *sugC*).

### 4.10. Reverse Transcription

(RT)-PCR verification: RT-PCR verification was performed to assess transcription of the LpqY-SugABC operon in Mtb wild-type, LpqY-SugABC^(−)^ knockout, and LpqY-SugABC^(+)^-complemented Mtb clones grown to mid-log phase (OD_600_ 0.4–0.6) in Middlebrook 7H9 medium. Total RNA was extracted using TRIzol reagent following the manufacturer’s protocol, including DNase I treatment to remove genomic DNA contamination, and RNA integrity and concentration were confirmed spectrophotometrically (A260/A280 > 1.8). First-strand cDNA synthesis was carried out using SuperScript IV Reverse Transcriptase with random hexamer primers, after which RT-PCR was performed using gene-specific primers for *Rv1234*, *lpqY*, *sugA*, *sugB*, and *sugC* genes, each yielding ~150 bp amplicons. Amplification was conducted under standardized cycling conditions (94 °C initial denaturation for 3 min; 30 cycles of 94 °C for 30 s, 60 °C for 30 s, and 72 °C for 1 min; and final extension at 72 °C for 3 min), and products were resolved by 1% agarose gel electrophoresis with ethidium bromide staining. Expression of 16S rRNA served as an endogenous control to verify cDNA quality and successful reverse transcription.

### 4.11. Macrophage Infection Assay with Mtb LpqY-SugABC Clones

Mtb wild-type, LpqY-SugABC^(+)^-complemented, and LpqY-SugABC^(−)^ knockout clones were grown to mid-log phase (OD_600_ 0.4–0.6) in Middlebrook 7H9 broth supplemented with 10% OADC, 0.05% Tween-80, and 0.5% glycerol at 37 °C with shaking at 200 rpm. Bacterial inocula were prepared as described above and used to infect THP-1 monolayers in 96-well plates at an MOI of 10 for 2 h at 37 °C and 5% CO_2_. After infection, extracellular bacteria were removed by washing three times with pre-warmed HBSS, and fresh RP10 medium was added; infection efficiency was verified at time zero by CFU recovery. Infected macrophages were then treated with isoniazid (INH, 2 μg/mL), rifampicin (RMP, 2 μg/mL), moxifloxacin (MXF, 1 μg/mL), or bedaquiline (TMC, 0.12 μg/mL), or left untreated as a control, and incubated under standard conditions. Intracellular bacterial survival was assessed at 1 h, 24 h, 48 h, 96 h, and 144 h post-infection by lysing macrophages with 0.1% Triton X-100, followed by serial dilution of lysates and plating on Middlebrook 7H10 agar for CFU enumeration to calculate bacterial survival over time.

### 4.12. ROS Sensitivity Assay for Compound Activity

*M. smegmatis* mc^2^155 (wild type), and *M. smegmatis* mc^2^155 ΔsugC and ΔlpqY mutants, as well as Mtb wild type and LpqY-SugABC^(−)^ mutant, were used for the hydrogen peroxide (H_2_O_2_)-susceptibility experiments and maintained on Middlebrook 7H10 agar. The single colonies were used to inoculate 10 mL Middlebrook 7H9 broth supplemented with 0.05% Tween 80 and 2.0% (*w*/*v*) glucose and cultured at 37 °C with shaking at 200 rpm for 48 h. After growth, cultures were harvested by centrifugation (5000× *g*, 15 min, room temperature), washed, and resuspended in 7H9 medium containing 0.05% Tween 80 and 0.02% (*w*/*v*) glucose to impose carbon limitation, and then normalized to an OD_600_ at 0.1 and incubated overnight at 37 °C with shaking (200 rpm). Next day, samples were adjusted to an OD_600_ of 1.0, H_2_O_2_ was added to final concentrations of 0.3–0.4% (*v*/*v*), and cultures were incubated for 10 min for *M. smegmatis* or 1 h for Mtb at 37 °C with shaking, after which cells were serially diluted ten-fold in 7H9 medium, and 4 µL of each dilution was spotted onto Middlebrook 7H9 agar plates supplemented with 2% (*w*/*v*) glucose; plates were incubated at 37 °C, and survival was assessed by the highest dilution showing visible growth compared to untreated controls.

To evaluate transporter-dependent effects on H_2_O_2_ sensitivity during compound treatment, the assay was performed as described above using Mtb wild type. Subinhibitory concentrations of compounds were added for 30 min before exposure to H_2_O_2_. Survival was assessed by spot assays and quantified by CFU enumeration. Results are presented as the fold-change in survival following H_2_O_2_ treatment in compound-treated relative to untreated Mtb cells.

### 4.13. In Vitro and In Situ Metabolic Labeling of Mtb

FITC-tre probe was prepared as previously described [28] at 10 mM in sterile phosphate-buffered saline (PBS; pH 7.4) and stored at −20 °C in light-protected containers until use. For labeling experiments, FITC-tre stock was diluted to a working concentration of 100 μM in either Middlebrook 7H9 medium for in vitro experiments or RP10 for macrophage infection experiments. The specificity of FITC-tre uptake by Mtb has been previously validated and confirmed to be highly selective for trehalose-metabolizing mycobacteria [28].

To demonstrate specific incorporation of FITC-tre in vitro via the LpqY-SugABC transporter system, Mtb wild-type, LpqY-SugABC^(+)^-complemented, and LpqY-SugABC^(−)^ knockout strains constitutively expressing tomato red protein [35] were inoculated into 10 mL of Middlebrook 7H9 medium at an OD_600_ of 0.5. FITC-tre was added to achieve a final concentration of 100 μM. As a negative control, Mtb wild type was cultured in identical conditions without FITC-tre supplementation. All cultures were incubated at 37 °C with orbital aeration (200 rpm) for 24 h. Following the 24 h incubation period, bacterial cultures were harvested by centrifugation at 5000× *g* for 15 min at 4 °C. Cell pellets were washed three times with ice-cold HBSS to remove excess probe and unincorporated FITC-tre. After washing, bacteria were fixed by incubation with 4% paraformaldehyde (PFA) in PBS for 20 min at room temperature with gentle agitation. Following fixation, cells were washed again with HBSS (3×) and resuspended in PBS at approximately 10^8^ CFU/mL for subsequent microscopic analysis. Fixed bacterial samples (10 μL) were mounted on glass slides (Superfrost Plus, Fisher Scientific, Pittsburgh, PA, USA) using 50 μL of ProLong Gold Antifade Mountant (Thermo Fisher Scientific, Waltham, MA, USA) and sealed with 22 mm × 22 mm coverslips (VWR, West Chester, PA, USA). Slides were allowed to cure at room temperature prior to imaging.

For intracellular Mtb metabolic labeling, THP-1 cells were infected with Mtb wild-type, LpqY-SugABC^(−)^ knockout, or LpqY-SugABC^(+)^-complemented strains, each constitutively expressing tomato red protein. Infections were performed at an MOI of 10 bacteria per cell for 2 h at 37 °C in a humidified 5% CO_2_ atmosphere. Following infection, cell monolayers were gently washed three times with ice-cold HBSS to remove extracellular bacteria. Next, fresh RP10 medium supplemented with FITC-tre at a final concentration of 100 μM was added to each well. Infected macrophages were incubated for an additional 24 h at 37 °C with 5% CO_2_ to permit intracellular bacterial growth and metabolic incorporation of FITC-tre. Infected THP-1 cell monolayers in culture dishes were gently washed three times with ice-cold HBSS and fixed by addition of 4% PFA in PBS for 20 min at room temperature with gentle rocking. Following fixation, cells were washed with PBS (3×) and permeabilized with 0.1% Triton X-100 in PBS for 5 min at room temperature. Cell nuclei were counterstained with 4′,6-diamidino-2-phenylindole (DAPI; 1 μg/mL in PBS) for 10 min at room temperature. Following nuclear staining, cells were washed with PBS (3×) and mounted using ProLong Gold Antifade Mountant. Mounted slides were allowed to cure at room temperature prior to imaging.

### 4.14. Fluorescence Microscopy

Fluorescence imaging was conducted using a Zeiss LSM 710 confocal laser scanning microscope (Carl Zeiss AG, Oberkochen, Germany) fitted with a 63× oil immersion objective (Plan-Apochromat, NA 1.40). Excitation was provided by 405 nm, 488 nm, and 561 nm laser lines for the DAPI, FITC, and tomato red channels, respectively, with emission collected through 410–480 nm, 500–550 nm, and 570–620 nm bandpass filters, respectively.

For quantitative analysis, at least 200 bacteria were analyzed per experimental condition from randomly selected fields in three independent biological experiments. Mean fluorescence intensity (MFI) of FITC labeling was quantified using ImageJ (version 1.54f, National Institutes of Health, Bethesda, MD, USA) after background subtraction. For intracellular experiments, colocalization between FITC-tre and tomato red fluorescence was assessed using ImageJ, and Manders’ overlap coefficients were calculated. The percentage of FITC-positive bacteria was determined using a predefined fluorescence threshold established from untreated controls. Data are presented as mean ± SD, and statistical comparisons were performed using one-way ANOVA with *p* < 0.05 considered statistically significant.

### 4.15. Compound Activity Validation via In Vitro Metabolic Labeling of Mtb

Mtb wild-type and LpqY-SugABC^(−)^ knockout strains were inoculated into 3 mL of Middlebrook 7H9 medium at an initial OD_600_ of 0.5. The tested compounds were added at subinhibitory concentrations 30 min prior to the addition of 100 μM FITC-tre. As a negative control, Mtb wild type and LpqY-SugABC^(−)^ were cultured in identical conditions with FITC-tre supplementation but no compounds added. All cultures were incubated at 37 °C with orbital aeration (200 rpm) for 24 h. Following incubation, bacterial cells were harvested by centrifugation at 5000× *g* for 15 min at 4 °C. Cell pellets were washed three times with HBSS to remove excess and unincorporated FITC-tre and then resuspended in HBSS. Fluorescence intensity was measured using a Tecan fluorometer with excitation and emission wavelengths of 485 nm and 525 nm, respectively.

### 4.16. Recombinant Expression and Purification of Mtb Trehalase

Mtb Rv2402 trehalase gene was amplified and cloned into the pET6xHN_C expression vector (Takara Bio, San Jose, CA) using the HindIII and EcoRI restriction sites. The gene was amplified with the primers Rv2402_F (5′-TTTTTTAAGCTTGCTCTATCGTCCAGTTCG-3′) and Rv2402_R (5′-TTTTTTGAATTCATGGGGGCGTTGGC-3′). The recombinant plasmid was transformed into *Escherichia coli* BL21(DE3) chemically competent cells (Takara Bio) and grown in Luria-Bertani (LB) medium supplemented with carbenicillin (50 µg/mL) at 37 °C with shaking (200 rpm) until cultures reached mid-log phase (OD_600_ of 0.6–0.8). Protein expression was induced by the addition of isopropyl β-D-1-thiogalactopyranoside (IPTG) to a final concentration of 0.5 mM, and cultures were incubated for 16–18 h at 18 °C with shaking (200 rpm) to enhance soluble protein production. Cells were harvested by centrifugation (5000× *g*, 15 min, 4 °C) and lysed by sonication on ice in lysis buffer (50 mM Tris-HCl, pH 8.0, 300 mM NaCl, 10 mM imidazole, 1 mM PMSF, and a protease inhibitor cocktail Sigma-Aldrich, St. Louis, MO, USA]), followed by centrifugation (5000× *g*, 30 min, 4 °C) to clarify the lysate. Recombinant Rv2402 was purified using Capturem His-tagged purification kit (Takara Bio) from the clarified lysate by immobilized metal affinity chromatography (IMAC) using Ni-NTA resin that selectively binds the 6 × HN tag, with bound protein eluted with 20 mM imidazole in the same buffer. Following IMAC elution, imidazole was removed by buffer exchange into the desired storage buffer using Zeba™ Spin Desalting Columns (Thermo Fisher Scientific, Waltham, MA, USA) according to the manufacturer’s instructions. Purified sample was analyzed by SDS-PAGE (12% gradient gel; Bio-Rad, Hercules, CA, USA) with Coomassie blue staining and imaged on a ChemiDoc imaging system (Bio-Rad), and protein concentration was determined using a BCA Protein Assay Kit (Pierce/Thermo Fisher Scientific).

### 4.17. Assessment of Trehalase Inhibition by Compounds

To quantify recombinant Rv2402 trehalase activity, we used a glucose oxidase/peroxidase (GAGO20, Sigma-Aldrich) colorimetric system via measurement of glucose released from trehalose hydrolysis. Briefly, the o-dianisidine reagent was reconstituted in 1.0 mL deionized water, and 0.8 mL was combined with 39.2 mL glucose oxidase/peroxidase reagent immediately prior to use. Glucose standards (0–0.02 mg/mL) were prepared by serial dilution in deionized water to generate a calibration curve, alongside reagent blanks and no-enzyme controls to account for background signal. The glucose standards were processed in parallel with experimental samples and used to establish the relationship between absorbance at 540 nm and known glucose concentrations. A standard curve was generated by plotting absorbance values against corresponding glucose concentrations, and the resulting linear regression equation was used to convert sample absorbance values into glucose concentrations. Glucose concentrations were determined from absorbance values using a glucose standard curve, and Rv2402 enzymatic activity was calculated based on glucose production. Trehalase activity was therefore quantified as the amount of glucose generated from trehalose hydrolysis under each experimental condition, with reduced glucose production indicating decreased enzymatic activity. For compound inhibition assays, purified Rv2402 trehalase (50 µg/mL final concentration) was pre-incubated with test compounds at subinhibitory concentrations for 30 min at 37 °C, where the enzyme + substrate + DMSO group served as the vehicle-only control. Reactions were initiated by adding trehalose substrate (5 mM final concentration) and incubated for 1 h at 37 °C. Reactions were terminated with 12 N sulfuric acid, followed by the addition of working assay reagent and incubation for 20 min at room temperature for color development. Absorbance was measured at 540 nm using a Tecan plate reader, and enzymatic activity in compound-treated samples was compared with untreated controls and expressed as a percentage of control activity. All conditions were tested in technical triplicate across two independent experiments, and raw absorbance values were background-subtracted using no-enzyme control wells.

### 4.18. Statistical Analysis

To demonstrate reproducibility, most experiments were performed with three technical replicates and repeated in three independent biological trials, unless otherwise specified in the corresponding figure legend. Statistical significance between experimental and control groups was assessed in GraphPad Prism (version 11.0) using one-way ANOVA with Dunnett’s post hoc multiple-comparison test for comparisons of multiple treatment groups against a single designated reference or control group, or two-way ANOVA with Tukey’s post hoc multiple-comparison test for time-course experiments involving comparisons among multiple bacterial strains at each time point. The specific test applied, number of independent biological replicates (n), and number of technical replicates are indicated in each figure legend. Significant *p*-values were assigned as * *p* < 0.05, ** *p* < 0.01, and *** *p* < 0.001.

## Figures and Tables

**Table 1:** List of active compounds identified in this study.

Cluster	Compound	Structure	Name
Aminothiazol	**59**	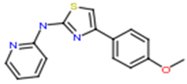	4-(4-methoxyphenyl)-N-pyridin-2-yl-1,3-thiazol-2-amine
Propanamide	**60**	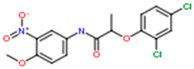	2-(2,4-dichlorophenoxy)-N-(4-methoxy-3-nitrophenyl)propanamide
Thiourea	**61**	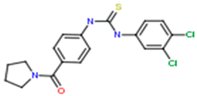	1-(3,4-dichlorophenyl)-3-[4-(pyrrolidine-1-carbonyl)phenyl]thiourea
Dicarboxylate	**62**	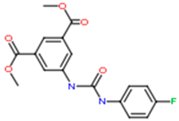	dimethyl 5-[(4-fluorophenyl)carbamoylamino]benzene-1,3-dicarboxylate
Propanamide	**66**	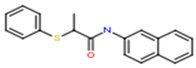	N-(naphthalen-2-yl)-2-(phenylsulfanyl)propanamide
Pentanamide	**67**	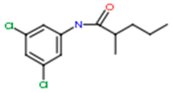	N-(3,5-dichlorophenyl)-2-methylpentanamide
Benzamide	**68**	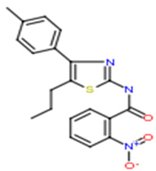	N-[4-(4-methylphenyl)-5-propyl-1,3-thiazol-2-yl]-2-nitrobenzamide
Glycinamide	**69**	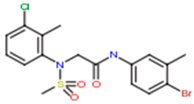	N-(4-bromo-3-methylphenyl)-2-(3-chloro-2-methyl-N-methylsulfonylanilino)acetamide
Thiourea	**72**	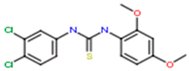	1-(3,4-dichlorophenyl)-3-(2,4-dimethoxyphenyl)thiourea
Carboxamide	**73**	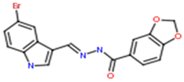	N-[(Z)-(5-bromo-1H-indol-3-yl)methylideneamino]-1,3-benzodioxole-5-carboxamide
Carboxamide	**74**	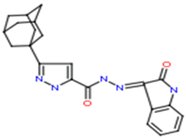	5-(1-adamantyl)-N-[(2-hydroxy-1H-indol-3-yl)imino]-1H-pyrazole-3-carboxamide
Benzimidazole	**75**	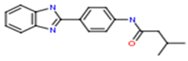	N-[4-(1H-benzimidazol-2-yl)phenyl]-3-methylbutanamide
Benzoate	**76**	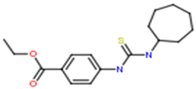	ethyl 4-(cycloheptylcarbamothioylamino)benzoate
Benzamide	**77**	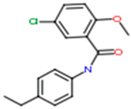	5-chloro-N-(4-ethylphenyl)-2-methoxybenzamide
Benzoxazole	**80**	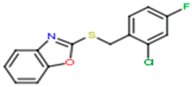	2-[(2-chloro-4-fluorophenyl)methylsulfanyl]-1,3-benzoxazole
Thiourea	**82**	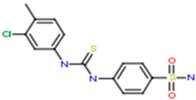	1-(3-chloro-4-methylphenyl)-3-(4-sulfamoylphenyl)thiourea
Acetamide	**83**	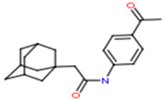	N-(4-acetylphenyl)-2-(1-adamantyl)acetamide
Oxadiazol	**86**	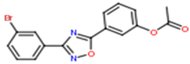	[3-[3-(3-bromophenyl)-1,2,4-oxadiazol-5-yl]phenyl] acetate
Benzamide	**88**	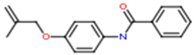	N-[4-(2-methylprop-2-enoxy)phenyl]benzamide
Benzamide	**89**	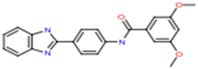	N-[4-(1H-benzimidazol-2-yl)phenyl]-3,5-dimethoxybenzamide
Carboxamide	**90**	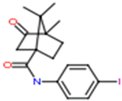	N-(4-iodophenyl)-4,7,7-trimethyl-3-oxobicyclo[2.2.1]heptane-1-carboxamide
Triazole	**92**	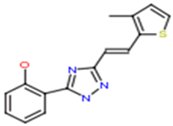	2-[5-[(E)-2-(3-methylthiophen-2-yl)ethenyl]-1H-1,2,4-triazol-3-yl]phenol
Thiourea	**95**	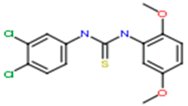	1-(3,4-dichlorophenyl)-3-(2,5-dimethoxyphenyl)thiourea

**Table 2:** Activity of hit compounds in vitro and in THP-1 macrophages.

Compounds	THP-1 Toxicity [μM]	A549 Toxicity [μM]	IC_50_ [μM] MM Trehalose
**59**	-	-	10
**60**	-	-	10
**61**	100	100	3
**62**	100	100	100
**66**	-	-	3
**67**	-	-	32
**68**	100	100	3
**69**	100	100	10
**72**	-	-	10
**73**	100	100	3
**74**	100	100	3
**75**	-	-	100
**76**	-	-	32
**77**	100	100	32
**80**	-	-	100
**82**	-	-	10
**83**	-	-	100
**86**	-	-	32
**88**	-	-	32
**89**	100	100	100
**90**	100	100	100
**92**	100	100	100
**95**	-	-	3

**Table 3:** ROS susceptibility in mycobacterial strains.

Species	Strain	H_2_O_2_ Concentration	Exposure Time	* Survival vs. WT
*M. smegmatis*	mc^2^155 (WT)	0.3%	10 min	1 (Baseline)
	ΔsugC	0.3%	10 min	~10-fold ↓
	ΔlpqY	0.3%	10 min	~15-fold ↓
*M. tuberculosis*	CDC1551 (WT)	0.4%	1 h	1 (Baseline)
	LpqY-SugABC^(–)^	0.4%	1 h	~20-fold ↓
	LpqY-SugABC^(+)^	0.4%	1 h	~1.5-fold ↓

* Data represent the mean fold-change in survival relative to wild type from three independent biological experiments (n = 3), each performed in triplicate technical replicates. ↓ Fold decrease relative to the wild type.

## Data Availability

The original contributions presented in this study are included in the article. Further inquiries can be directed to the corresponding author.
